# *Yersinia enterocolitica* O:3 Outer Membrane Vesicles as a Platform for Complement Activation

**DOI:** 10.1002/jev2.70270

**Published:** 2026-04-17

**Authors:** Cédric Battaglino, Iryna Bodnaruk, Paula Czyszczoń, Mikael Skurnik, Beata Filip‐Psurska, Dariusz Jarych, Anna Maciejewska, Mariusz Gadzinowski, Kamil Malik, Izabela Potocka, Paweł Migdał, Roksana Kruszakin, Maciej Cedzyński, Katarzyna Kasperkiewicz, Jolanta Lukasiewicz, Anna S. Świerzko

**Affiliations:** ^1^ Institute of Medical Biology Polish Academy of Sciences Lodz Poland; ^2^ BioMedChem Doctoral School of University of Lodz and Lodz Institutes of the Polish Academy of Sciences Lodz Poland; ^3^ Institute of Biology, Biotechnology and Environmental Protection Faculty of Natural Sciences University of Silesia Katowice Poland; ^4^ Ludwik Hirszfeld Institute of Immunology and Experimental Therapy Polish Academy of Sciences Wroclaw Poland; ^5^ Department of Bacteriology and Immunology, Human Microbiome Research Program, Faculty of Medicine University of Helsinki Helsinki Finland; ^6^ Center of Molecular and Macromolecular Studies Polish Academy of Sciences Lodz Poland

**Keywords:** biodistribution, complement, lipopolysaccharide (LPS), mannose‐binding lectin (MBL), outer membrane vesicles (OMVs), systemic inflammatory response syndrome (SIRS), *Yersinia*

## Abstract

Lipopolysaccharide (LPS) is the major component of the outer membrane vesicles (OMVs) of Gram‐negative bacteria. We hypothesized that OMVs‐induced complement activation contributes to the development of systemic inflammatory response syndrome (SIRS). *Yersinia enterocolitica* O:3 (YeO3) variants synthesizing LPS of various chemotypes (S, Ra, Rd1, Re) were used as model microorganisms. OMVs activated complement more potently than parental bacteria or homologous LPS, independently of chemotype. A high molecular weight polysaccharide fraction, distinct from LPS, was recognized by serum mannose‐binding lectin (MBL). *In vivo* experiments demonstrated that complement depletion weakened the hallmarks of OMVs‐induced SIRS in mice. LPS chemotype affected the biodistribution of OMVs and long O‐specific polysaccharide protected them from clearance. Chemotype influenced OMVs secretion with their highest release by bacteria with LPS reduced to the inner core and lipid A (Rd1). The shift from environmental to host's temperature stimulated secretion of smaller OMVs, with less‐toxic, tetra‐acyl lipid A. Our data are consistent with a contribution of OMVs to *Yersinia* pathogenicity through complement activation. Their potency as complex virulence factor is influenced by size, length of oligo‐/polysaccharide chain, and lipid A form. This study comprehensively characterizes OMV‐complement interactions in YeO3, extending the knowledge of mechanisms previously established for other Gram‐negative bacteria.

## Graphical abstract

Basic characteristics and biological activity of outer membrane vesicles (OMVs) secreted by *Yersinia enterocolitica* O:3 (YeO3), depending on LPS chemotype and growth temperature.

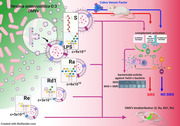

## 1 Introduction

Both eukaryotic and prokaryotic cells release extracellular vesicles, differing in size, envelope structure and composition. In Gram‐negative bacteria, they originate from the outer membrane (OM) and therefore are termed outer membrane vesicles (OMVs). OMVs contain a variety of proteins, phospholipids, peptidoglycans (PG), lipopolysaccharides (LPS, endotoxin) and encapsulated periplasm. The constitution of the OMV outer membrane may differ from that of the parental strain OM (reviewed by Magaña et al. 2023). Moreover, OMVs may carry specific cargoes depending on the bacterial origin and environmental factors.

The function of OMVs is multifaceted, ranging from their involvement in the promotion of infection to their role as a potent immunogen that stimulates the immune response to the pathogen (reviewed by Roier et al. 2016). Vesicles have been found in clinical samples of body fluids and tissue sections (Craven et al. 1980; deVoe and Gilchrist 1975; Perez Vidakovics et al. 2010; Ren et al. 2012; Schaack et al. 2022; Stephens et al. 1982). As evidenced in a murine model, OMVs are distributed within the body (Jang et al. 2015) and their *i.p*. injection may lead to the development of symptoms characteristic of systemic inflammatory response syndrome (SIRS)/sepsis (Park et al. 2010). However, no *in vivo* data concerning involvement of the OMV‐complement interplay process have been published so far.

Bacterial OMVs surface antigens, especially LPS and various membrane proteins, have potent pathogen‐associated molecular patterns (PAMPs) and can activate the complement system *via* alternative (AP), classical (CP) and/or lectin (LP) pathways.

Knowledge concerning OMV‐induced complement activation and its consequences is still rather scarce. Native or LPS‐depleted OMVs as well as whole cells of *Neisseria meningitidis* were more potent complement activators compared with purified LPS (Bjerre et al. 2002). Although AP was recognized as the main player, participation of CP/LP was not excluded. Blebs from serum‐resistant gonococcal strains had enhanced ability to bind and remove cell‐targeted bactericidal factors (Pettit and Judd 1992). Tan et al. (2007) demonstrated that OMVs isolated from *Moraxella catarrhalis* carry surface proteins UspA1 and UspA2, able to bind C3 and contribute to serum resistance. Interestingly, pre‐incubation of normal human serum (NHS) with *M. catarrhalis* OMVs significantly decreased also its bactericidal activity against *Haemophilus influenzae*.

The secretion of OMVs is associated with LPS release from bacterial cells (Dardelle et al. 2024). LPS, is one of the main components of Gram‐negative bacteria OM, covering approximately 70% of the cell surface. LPS, by contributing to development of sepsis/SIRS symptoms, is considered an important virulence factor. Natural LPS is a mixture of molecules consisting of lipid A substituted with the core oligosaccharide and O‐specific polysaccharide (OPS) chains of different lengths. The shortest substitution in the Re chemotype is limited to the 3‐deoxy‐D‐*manno*‐octulosonic acid (Kdo) residues (lipid A‐Kdo). The Rd chemotype contains additionally the heptose (Hep) residues of the inner core (lipid A‐IC), while LPS of the Ra chemotype contains the complete (inner + outer) core (lipid A‐IC‐OC). The longest endotoxin molecules of S chemotype in most bacterial species represent lipid A‐IC‐OC‐OPS structure (Rietschel et al. 1994). The LPS heterogeneity is increased by the different fatty acid, phosphate group (P), phosphoethanolamine (PEtn), and 4‐amino‐4‐deoxy‐α‐L‐arabinose (α‐L‐Ara4N) substitutions of the lipid A carbohydrate backbone and/or IC.

Whereas the lipid A part of LPS is its toxic principle, responsible for immune cell activation, the polysaccharide part contributes to the evasion of host immunity, modifies endocytosis and intracellular bacterial survival rate, and induces production of specific antibodies (reviewed by Maldonado et al. 2016). It furthermore may be involved in the induction of an anaphylactoid reaction associated with rapid death of mice (Świerzko et al. 2003; Zhao et al. 2002).

Both poly‐ and oligosaccharide parts as well as lipid A can induce complement system activation *via* AP, CP and/or LP (Galanos et al. 1971; Loos et al. 1974; Man‐Kupisinska et al. 2018; Morrison and Kline 1977; Roumenina et al. 2008; Swierzko et al. 2012).

Although complement generally contributes to the elimination of pathogens, its uncontrolled activation may lead to adverse effects, including severe symptoms associated with systemic inflammation. Therefore, its inhibition is a potential therapeutic strategy for treatment of SIRS/sepsis (Karasu et al. 2019).

To investigate LPS‐dependent induction of complement activation by OMVs and its possible role in SIRS development, we used *Yersinia enterocolitica* O:3 (YeO3) as a model bacterium. It is usually an aetiological agent of self‐limited gastroenteritis, but it may also cause sepsis, with a mortality rate above 50%. Its unique features include the ability to multiply within a very broad temperature range (from < 4°C to > 40°C) and the temperature‐regulated expression of its virulence factors (including LPS) suggesting environment‐shift induced phenotypic changes.

**FIGURE 1:**
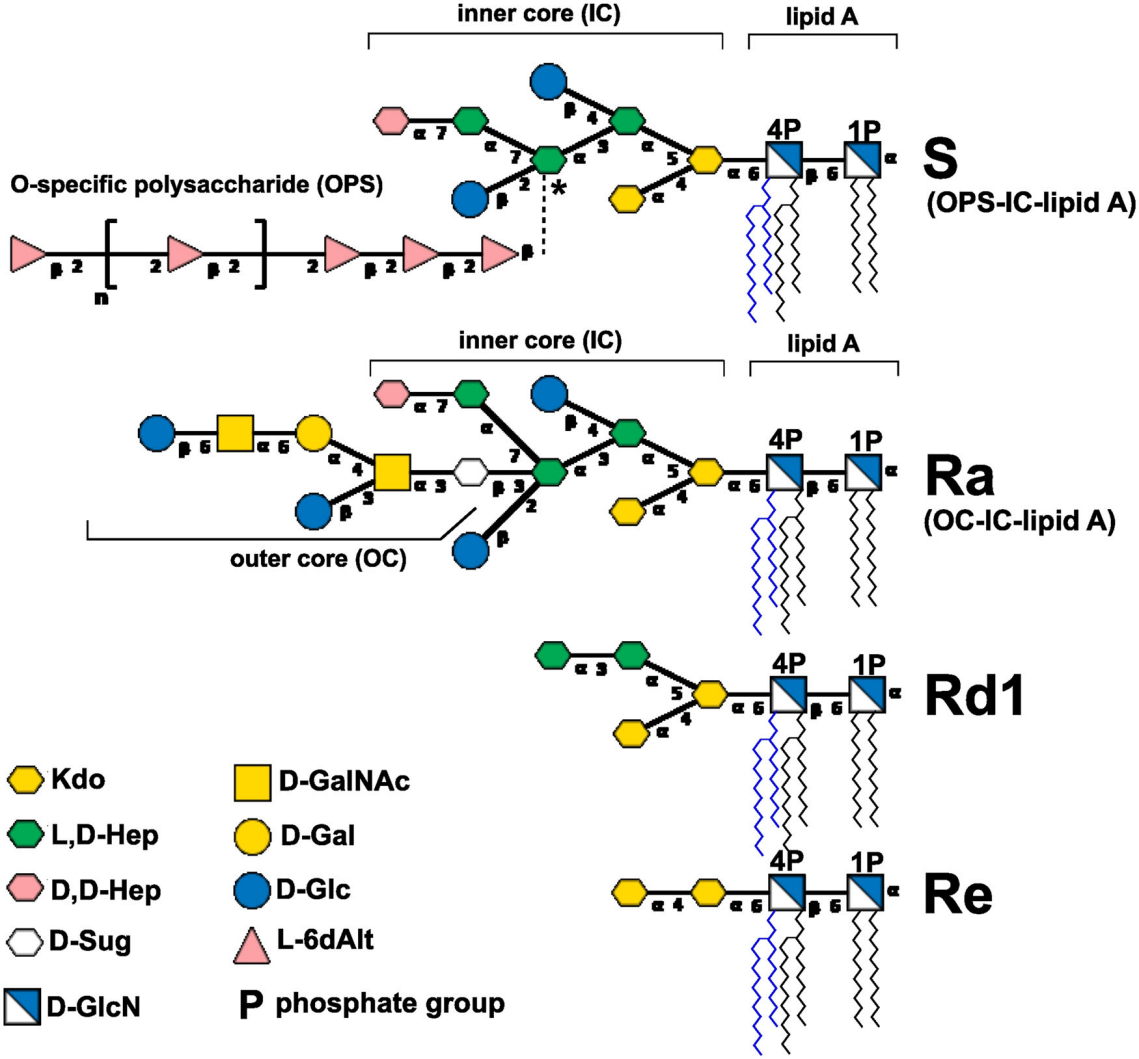
Schematic representation of *Yersinia enterocolitica* O:3 LPS structure with S, Ra, Rd1, and Re chemotypes (Muszyński et al. 2013; Pinta et al. 2009). The asterisk (*) indicates the putative OPS‐attachment residue of the inner core (IC). All sugars were pyranoses. L,D‐Hep, L‐*glycero*‐α‐D‐*manno*‐heptopyranose; D,D‐Hep, D‐*glycero*‐α‐D‐*manno*‐heptopyranose; Kdo, 3‐deoxy‐α‐D‐*manno*‐oct‐2‐ulopyranosonic acid; D‐Glc, β‐D‐glucopyranose; D‐Gal, α‐D‐galactopyranose; GalNAc, 2‐acetamido‐2‐deoxy‐α‐D‐galactopyranose; D‐Sug, 2‐acetamido‐2,6‐dideoxy‐δ‐D‐*xylo*‐hex‐4‐ulopyranose; L‐6dAlt, 6‐deoxy‐β‐L‐altropyranose. The sugars contained within the square brackets are from the repeating units of the O‐specific polysaccharide (OPS). The Symbol Nomenclature for Glycans (SNFG) was used for drawing LPS structures (Neelamegham et al. 2019; Varki et al. 2015). Lipid A region indicates major tetra‐ (black acyl chains) and hexaacylated (black and blue acyl chains) forms characteristic for bacteria culture at 37°C.

Moreover, *Yersinia* sp. bacteria synthesize LPS arranged differently from the general pattern described above. It is a mixture of lipid A‐IC molecules substituted either with long OPS (lipid A‐IC‐OPS, chemotype S) or with shorter OC (lipid A‐IC‐OC, chemotype Ra), but do not contain OC and OPS together, as in the majority of Gram‐negative bacteria (Figure 1). The YeO3 OPS is a homopolymer of β‐1,2‐linked 6‐deoxy‐L‐altrose [→2‐β‐L‐6dAlt] (Muszyński et al. 2013; Pinta et al. 2009). The structure of lipid A was reported for a few *Y. enterocolitica* serotypes including enteropathogenic O:3, O:8 and O:9 (Aussel et al. 2000; Fernandez‐Carrasco et al. 2025; Guo et al. 1998; Oertelt et al. 2001). *Yersinia* shows more variation in its lipid A structure in comparison with lipids A of *E. coli*, including substitution by secondary linked acyl chains: dodecanoic (12:0) at 3’ position and tetradecanoic (14:0) or *cis*‐hexadec‐9‐enoic acid (16:1) at the 2' position. The lipid A of Ye contains the β‐d‐Glc*p*N4P‐(1→6)‐α‐d‐Glc*p*N1P disaccharide substituted with acyl residues and α‐l‐Ara4N. Its acylation pattern is dependent on the growth temperature (Pérez‐Gutiérrez et al. 2010; Rebeil et al. 2004; Reinés et al. 2012a) and is associated with the decrease of acyl groups from 6–7 acyl chains at 21°C (hexa‐ and heptaacylated) to 4–6 acyl chains at 37°C (tetra‐ and hexaacylated) and reduced potential to immune cell stimulation, including induction of cytokines (Rebeil et al. 2004). Moreover, pyrophosphate groups (PP) and Etn were not reported for *Y. enterocolitica* lipids A. Such a unique LPS arrangement facilitated the isolation or engineering of OPS‐ or OC‐deficient variants and allowed testing of LPS activity associated with these regions separately (Biedzka‐Sarek et al. 2005; Kasperkiewicz et al. 2015).

As the composition of YeO3 LPS is affected by growth temperature (with longer OPS chains formation at lower temperature), this bacterium serves as a suitable model for determining the biological consequences of temperature shift‐induced structural modifications of OMVs‐exposed LPS. In addition, it is also useful for investigation of the interaction of OMVs with serum proteins (including factors of the complement cascade) and phages.

Our main goal was to analyse the contribution of OMV‐induced complement activation to the development of SIRS. We focused on YeO3 variants sensitive to complement lytic activity due to the loss of virulence plasmid pYV, synthesizing LPS of various chemotypes (Biedzka‐Sarek et al. 2008; Kirjavainen et al. 2008). We analyzed the ability of OMVs to activate complement *in vitro* as well as the impact of OMVs‐induced complement activation on the development of SIRS hallmarks *in vivo*. Moreover, we studied the influence of the LPS chemotype on the biodistribution and clearance rate of OMVs in mice. We investigated also the potency of OMVs as virulence factors in relation to their secretion, size, LPS chemotype and lipid A composition. Our findings permitted a more precise assessment of the significance of OMVs in pathogen‐host interactions, their role in both local and systemic responses, and the molecular basis of those relationships.

## 2 Materials and Methods

### 2.1 Growth Conditions of Bacterial Strains and Isolation of OMVs

The YeO3 strains used in this study are listed in Table 1. Structures of corresponding LPSs are given in Figure 1. Bacteria were grown aerobically at 37°C, 22°C or 4°C in LB medium (BTL, Poland), to late logarithmic phase (OD_600_ = 0.8–1.0). Ten passages at given temperature were performed before starting cultivation of bacteria for OMVs isolation. Cells were then pelleted at 6000 × g, heat inactivated (60°C, 1 h), washed with deionised water and lyophilised. Culture supernatant was vacuum‐filtered (0.22 µm), tested for sterility by plating on LB‐agar and used for testing of inhibition of bactericidal serum activity or ultracentrifuged (Optima ultracentrifuge, Beckman, USA) using a 45Ti rotor at 100 000 × g (30 000 rpm) for 2 h, at 4°C. The OMV pellet was washed 2x with phosphate buffered saline (PBS) (Gibco, USA), filtered (0.22 µm), tested for sterility and stored at −20°C (“crude OMVs”). Alternatively, cell‐free culture supernatants were concentrated using a 100 kDa membrane (Sartorius, Germany) and then ultracentrifuged as described above. The OMV preparation obtained was described as “>100 kDa OMVs”. To get “density gradient purified OMVs”, >100 kDa OMV preparations were further purified using Optiprep (Merck, Germany) density gradient ultracentrifugation (according to Kohl et al. 2018). The OMVs accumulated at the 15%–35% iodixanol interface (YeS‐c_37°C – 20%–30%, YeS‐c_22°C – 15%–30%, YeS‐c_4°C – 15%–30%, YeRa‐c_37°C – 20%–30%, YeRd1‐c_37°C – 15%–30% and YeRe‐c_37°C – 20%–30%). For long term storage OMVs were kept at −80°C.

**TABLE 1:** Bacterial strains used in the study.

LPS chemotype	*Y. enterocolitica* O:3 strain	Description	References
**S** (LA‐IC‐OPS & LA‐IC‐OC)	YeO3‐c 6471/76‐c (YeS‐c)	virulence plasmid‐cured derivative of 6471/76 (serotype O:3, patient stool isolate, wild type strain)	Skurnik et al., 1984
**Ra** (LA‐IC‐OC)	YeO3‐c‐R1 (YeRa‐c)	spontaneous rough derivative of strain 6471/76‐c	Al‐Hendy et al. 1992
**Rd1** (LA‐4/8IC)	YeO3‐c‐R1‐M196 (YeRd1‐c)	*galU*::Cat‐Mu. transposon insertion derivative of YeO:3‐c‐R1, ClmR	Noszczyńska et al. 2015
**Re** (LA‐2/8IC)	YeO3‐c‐R1‐M205 (YeRe‐c)	*hldE*::Cat‐Mu. transposon insertion derivative of YeO:3‐c‐R1, ClmR	
**S** (LA‐IC‐OPS & LA‐IC‐OC)	YeO3‐Ail 6471/76‐Ail	*∆ail*::Km‐GenBlock, Km^r^ derivate of 6471/76	Biedzka‐Sarek et al. 2005
**S** (LA‐IC‐OPS & LA‐IC‐OC)	YeO3‐c‐Ail 6471/76‐c‐Ail	*∆ail*::Km‐GenBlock, Km^r^ derivate of 6471/76‐c	

Abbreviations: LA, lipid A; IC, inner core; OC, outer core; OPS, O‐specific polysaccharide.

If necessary, OMVs were incubated with 20 U of DNase I (ThermoFisher Scientific, USA) for 60 min at 37°C or treated with Proteinase K (4 µg, 1 h, 60°C) (ThermoFisher Scientific).

### 2.2 Determination of OMV Concentrations and Dimensions

The concentration and size of isolated OMVs were determined using nanoparticle tracking analysis (NTA) system (NanoSight NS300 device, Malvern Panalytical, UK) with Malvern NTA analysing software (version 3.4). Samples containing OMVs were diluted in filtered PBS for analysis. NTA was performed by capturing of three 45 s movies at camera level 13 at 25°C and detection threshold 3. The average of the three records was calculated.

### 2.3 Transmission Electron Microscopy (TEM)

OMVs samples (4 µL, room temperature) were applied onto the formvar supported copper grid (Agar Scientific, Great Britain) and incubated for evaporation (or for 10–15 min and excess of liquid was blotted from the grid). Samples were negatively stained with 2% uranyl acetate (MicroShop, Poland) for 1 min. subsequently. Grids were air dried and imaged with JEOL JEM F‐1200 (JEOL, Japan) transmission electron microscope operating at 80 kV.

### 2.4 Scanning Electron Microscopy (SEM)

The detection of OMVs in bacterial cultures and analysis of their size were performed with SEM. Droplets of the bacterial suspension were pipetted onto a small poly‐L‐lysine‐coated slides. The slides were immersed in a fixative solution (2.5% glutaraldehyde in 0.1 M phosphate buffer, at pH 7.2) and incubated overnight at 4°C. The samples were then washed with phosphate buffer, dehydrated with a graded ethanol series, dried by the critical point drying method (Leica EM CPD300, Leica Microsystems, Austria), mounted on aluminium stubs, and sputter coated with a 15 nm layer of gold (Safematic CCU‐010 HV compact coating unit, Safematic GmbH, Switzerland). Observations were carried out with a field emission scanning electron microscope UHR FE‐SEM Hitachi SU8010 (Hitachi High‐Technology Corporation, Japan) in secondary electron mode at accelerating voltages of 5 kV and 10 kV. OMV diameters were measured on the captured images using the in‐built software (Hitachi PC‐SEM Data Manager 2.7).

### 2.5 OPS and ECA Detection on OMVs Surface

To detect YeO3 enterobacterial common antigen (ECA), MaxiSorp U96 plates (Nunc, Denmark) were coated with OMVs. After blocking, mAb 898 recognising the ECA (kindly provided by Prof. D. Bitter‐Suermann, Institute of Medical Microbiology, Hannover, Germany) and corresponding HRP‐labeled secondary antibodies were used. The substrate for peroxidase was 2,2'‐azino‐bis(3‐ethylbenzothiazoline‐6‐sulfonic acid) (ABTS) (Sigma‐Aldrich, USA). TomA6 (Al‐Hendy et al. 1991) mAb which recognizes YeO3 OPS homopolymer of β‐1,2‐linked 6‐deoxy‐l‐altrose was used for immunodetection of OPS in OMVs in Western blot.

### 2.6 Interaction of YeO3 Specific Bacteriophages With OMVs

To test the ability of isolated OMVs from YeO3 of S chemotype (YeS) to bind YeO3‐specific bacteriophages, phage φYeO3‐12 [10^4^ plaque forming units (PFU)] (Pajunen et al. 2000) was preincubated with 35, 70, 140, 280 and 560 ng of OMVs or LPS solution (50 µL) or 50 µL PBS and then 10 µL of the mixture was plated on *Y. enterocolitica* O:3 lawn culture. The formation of plaque of lysis was examined after 18 h incubation at 37°C.

### 2.7 LPS Isolation

The LPS from smooth *Y. enterocolitica* O:3 strain was isolated by a hot phenol/water method according to Westphal and Jann (1965), whereas the LPSs of the rough strains were isolated by hot phenol/water extraction followed by the phenol/chloroform/petroleum ether method (Galanos et al. 1969).

### 2.8 Structural Analysis of Cell‐Derived LPS and OMV‐Derived LPS

To compare structures of the OMV‐derived LPS with cell‐derived LPS, density gradient purified OMVs (23–71 µg of total protein) and cell‐derived LPSs (100 µg) were hydrolyzed with 1.5 % acetic acid (45 min, 100°C) and centrifuged (14 000 × g, 20 min, 4°C) to isolate the lipid fraction (including lipid A) and soluble supernatant containing OPS, IC‐OC, and IC (Micoli et al. 2022). Soluble supernatants were fractionated by size‐exclusion chromatography on a TSKgelG2500PW column (7.5 × 60 cm, flow: 1 mL/min) (Tosoh Bioscience, Belgium) and equilibrated with 0.05 M acetic acid. The fractions (1 mL) obtained and sediments containing lipids A were freeze‐dried and analyzed by matrix‐assisted laser‐desorption/ionization time‐of‐flight (MALDI‐TOF) mass spectrometry (MS) or by nuclear magnetic resonance (NMR) spectroscopy.

### 2.9 Mass Spectrometry

MALDI‐TOF MS was carried out on a MALDI‐TOF/TOF Bruker ultrafleXtreme spectrometer (Bruker BioSpin GmbH, Germany). 9H‐pyrido[3,4‐b]indole [1% in a 1:1 acetonitrile/water mixture (v/v)] was used as matrix for the analysis of lipid A sediments in negative ion mode as previously described (Lukasiewicz et al. 2006). Lipids A (sediments obtained after LPS and OMV hydrolysis) were dissolved in chloroform/isopropanol/water (5:3:0.25) and mixed with the matrix in the 1:1 ratio (v/v). ChemBioDraw Ultra v.14.0.0.117 (PerkinElmer, USA) was used for drawing of lipid A structure and *m/z* calculations.

### 2.10 NMR Spectroscopy

The OPSs isolated from LPS and OMVs were analyzed by NMR spectroscopy. The ^1^H NMR spectra were obtained at 298 K using on a Bruker NMR AVANCE III 600 MHz (Bruker BioSpin GmbH, Germany) spectrometer equipped with a 5 mm QCI cryoprobe with *z*‐gradient and 3 mm NMR tubes. For acquiring and processing standard Bruker software was used.

### 2.11 Serum Bactericidal Activity Assay

To test the ability of OMVs to affect antibacterial serum activity, the overnight bacterial culture of pYV‐cured *Y. enterocolitica* O:3 (YeS‐c) was refreshed for 2 h at 37°C in high‐glucose DMEM (Gibco, USA) medium. Normal (active) or inactivated (56°C, 30 min) pooled human complement serum (NHS) (Innovative Research, USA) was diluted 10‐fold in a mixture (1:1) of sterile LB medium and PBS or mixture (1:1) of 0.22 µm filter‐sterilized culture supernatant (as a source of OMVs) and PBS, and incubated for 2 h, at 4°C. Next, 10 µL of bacterial suspension was added and the tubes were incubated for 30 min at 37°C (water bath). Then, 10 µL aliquots of serially diluted (10^−1^ to 10^−6^) samples were spotted on LB agar plates and incubated at 37°C overnight to observe the survival rate of the bacteria. The higher the dilution at which bacterial growth was observed, the higher the bactericidal serum activity.

### 2.12 Complement Activation *in Vitro* (ELISA)

To test OMV‐induced complement activation, MaxiSorp U96 plates (Nunc) were coated with 50 ng/well of isolated OMVs, heat‐inactivated bacteria or LPS and incubated with NHS diluted in veronal buffer supplemented with calcium and magnesium (1.8 mM sodium barbital, 3.1 mM barbituric acid, 0.15 mM CaCl_2_, 0.5 mM MgCl_2_, 0.1% gelatine, pH 7.4). To distinguish between complement activation *via* the CP/LP or AP, serum was treated with 5 mM EDTA, 5 mM EGTA or 5 mM EGTA supplemented with Mg^2+^. Plates were coated with 10^8^ of OMVs/well. After incubation for 1 h at 37°C, the products of complement activation were detected using polyclonal rabbit anti‐human C3c (Dako, Denmark) or rabbit anti‐human C4c (Dako) antibodies and HRP‐conjugated anti‐rabbit Abs (Dako). For terminal complement complex (TCC) detection, anti‐human C9 mAb (clone aE11, Hycult, The Netherlands) and anti‐mouse secondary antibodies were used. To inhibit classical pathway activity, while keeping LP functional, the serum was diluted in a high ionic strength MBL‐binding buffer (20 mM Tris/HCl, 1 M NaCl, 0.5 % BSA, pH 7.4) (Petersen et al. 2001) but with lower CaCl_2_ concentration (0.15 mM). MBL‐deficient serum (at 1:3000 dilution) was used as exogenous source of C4.

### 2.13 MBL Binding to OMVs (ELISA)

To test human MBL binding to OMVs, MaxiSorp U96 plates (Nunc) were coated with OMVs, bacteria or LPS as described above and incubated (4°C, overnight) with high‐MBL NHS (MBL level: 2 µg/mL) diluted in 1% BSA/imidazole buffer (40 mM imidazole, 1.25 M NaCl, 50 mM CaCl_2_, pH 7.8). The bound proteins were detected with murine antibodies against human MBL (HYB 131‐01, Statens Serum Institute Denmark) and anti‐mouse Ig‐HRP (Dako). To test the specificity of reaction, 5 mM EDTA was used to block lectin/sugar interaction.

To test the murine MBL‐A and MBL‐C interaction with OMVs, microtiter plates were coated with 10^8^ OMV particles/well. C57BL/6 serum was used as a source of MBLs. After overnight incubation at 4°C, the bound proteins were detected with specific rat monoclonal antibodies against MBL‐A and MBL‐C (clones 8G6 and 16A8, respectively, both purchased from Hycult) and goat anti‐rat Ig‐HRP (Dako).

### 2.14 Membrane‐Immobilized LPS Mild Acid Hydrolysis (Western Blot)

For mild hydrolysis of LPS, a method described by Pantophlet et al. (1997) was used. Briefly, bacterial cells, OMVs and LPSs were subjected to SDS‐PAGE and transferred to nitrocellulose. The membrane was next incubated at 100°C for 30 min in 1% acetic acid, washed extensively with imidazole buffer and next incubated with high‐MBL NHS and anti‐MBL antibody as described above. To detect lipid A exposed in the result of LPS hydrolysis, the specific mAb, A6 (kindly provided by prof. H. Brade, Research Center Borstel, Germany) and corresponding secondary antibody were used. Pierce ECL Western Blotting Substrate (Thermo Scientific) was used for blot development.

### 2.15 OMV Activity *in Vivo*

C57BL/6 male or BALB/c female mice (Charles River Laboratories, Animalab, Poland) were maintained in individual ventilated cages, provided sterile food and water *ad libitum*, and were used for experiments at the age of 6–8 weeks. All experiments were approved by the Local Ethical Committee for Animal Experiments with the Use Laboratory Animals, Wroclaw, Poland (permission numbers 049/2023/P1 and 021/2024).

#### 2.15.1 SIRS Induction

C57BL/6 male mice were *i.p*. treated with YeS‐c_37°C OMVs (>100 kDa OMVs, 15 µg of total protein/mice). The dose of OMVs was based on the protocol proposed by Jang et al. (2015). In preliminary experiments, it was confirmed that the dose of 15 µg of OMVs is not lethal, but that it affects blood cell count and induces changes in the expression of pro‐inflammatory cytokine genes. As a control, mice were injected with 0.9% NaCl. Depletion of complement was achieved as described by Ajona et al. (2007). Mice were treated *i.p*. with 3.5 U of *Naja naja kaouthia* cobra venom factor (CVF) (Quidel, USA) in 100 µL of PBS at 28, 24, and 4 h before injection of YeS_37°C OMVs. Depletion of functional C3 from serum was verified in Western blot [acc. to Younger et al. (2003)], using goat antibodies against mouse C3 (MP Biomedicals, USA), HRP‐conjugated anti‐goat Ig (Dako) for detection of intact C3 α‐chain and ECL detection system. Mice were euthanized 6 or 12 h (T6, T12) after OMVs injection. The blood was collected into tubes containing either EDTA or no chelator, to isolate the serum. Blood cell morphology was analyzed using the Mythic 18 haematology analyser (PZ Cormay S.A, Poland). Liver tissue samples were submerged in RNAlater (ThermoFisher Scientific) and after overnight incubation at 4°C, stored at –80°C.

#### 2.15.2 *in Vivo* Biodistribution Imaging

To analyse LPS chemotype‐dependent OMV distribution in mice, DiD fluorescence dye‐ (Vybrant^TM^DiD Cell‐labeling solution, ThermoFischer Scientific) labeled OMVs (crude OMVs, 15 µg) were injected intraperitoneally to female BALB/c mice according to Jang et al. (2015). OMVs were labelled with 5% (v/v) DiD by incubating at 37°C for 30 min. Unbound dye was removed using 30 kDa centrifugal filters (Sartorius). 0.9% NaCl‐ as well as DiD‐0.9% NaCl‐treated mice were used as controls. Mice were sacrificed after 3 h, 6 h, 12 h or 24 h (T3–T24). The fluorescence in the whole body as well as in isolated organs (liver, lung, spleen, kidney, and lymph nodes) was acquired by Carestream In‐Vivo FX Pro apparatus (Bruker, Germany) and analyzed with Bruker Molecular Imaging Software v.7.1.3.20550. The reader settings were as follows: excitation at 630 nm, emission at 750 nm, FOV 120, f‐stop 2.5, exposition 20 s. Data were displayed as absolute fluorescence (photons(P)/s/mm^2^). The fluorescence of 50 µL of blood (taken into tubes with EDTA) or organs was analyzed with Synergy H4 Hybrid Reader (Biotek, USA) (ex./em. 630 nm/675 nm).

### 2.16 RT PCR Analysis of Gene Expression

To determine hepatic expression of pro‐inflammatory genes after excision of the liver, the total RNA was isolated using the RNeasy Mini Kit (Qiagen, USA). High‐Capacity cDNA Reverse Transcription Kit (Applied Biosystems, ThermoFisher Scientific) was used for reverse transcription. The RT‐PCR analysis was performed using TaqMan Genotyping Master Mix (Applied Biosystems, ThermoFisher Scientific) and primers listed in Table 2. The data were normalized based on the mean expression of two mRNAs in each sample (HPRT and BM2 genes).

**TABLE 2:** The sequences of primers for determination of expression of genes coding mouse cytokines and C3.

Gene	Primer forward	Primer Reverse	References
*IL1B*	GAAATGCCACCTTTTGACAGTG	TGGATGCTCTCATCAGGACAG	Liu et al. 2018
*IL6*	CTGATGCTGGTGACAACCAC	CAGAATTGCCATTGCACAAC	Crocker et al. 2023
*TNF*	CCTGTAGCCCACGTCGTA	GGGAGTAGACAAGGTACAACCC	Liu et al. 2018
*C3*	ACTGTGGACAACAACCTACTGC	GCATGTTCGTAAAAGGCTCGG	Crocker et al. 2023
*HPRTa*	TGTTACCAACTGGGACGACA	GGGGTGTTGAAGGTCTCAAA	Souza et al. 2024
*B2M*	TCTCACTGACCGGCCTGTAT	GTATGTTCGGCTTCCCATTC	

### 2.17 MBL‐A and MBL‐C Detection in Mouse Serum and Bile (ELISA)

MaxiSorp U96 plates were coated with yeast mannan (Sigma‐Aldrich). After incubation with serum (diluted 500 × or 2000 ×, depending on protein to be detected) or bile (diluted 10 × in 1% BSA/imidazole buffer (4°C overnight), the bound proteins were detected using rat anti‐MBL‐A (8G6, Hycult), ‐MBL‐C (16A8, Hycult) and goat anti‐rat Ig‐HRP (Dako) antibodies.

### 2.18 Statistical Analysis

Graphical data were analyzed with GraphPad Prism 9 and presented as mean. Kruskal‐Wallis one‐way analysis of variance (ANOVA) with Dunn's post‐hoc test was used to determine statistical significance among multiple groups with one independent variable. When two independent groups were compared, non‐parametric Mann‐Whitney *U* test was used. Statistically significant differences were established by a *p‐*value < 0.05. The number of repeats is given in the figure or table captions.

## 3 Results

The YeO3 (S, Ra, Rd1, Re) OMVs were isolated from cultures grown at 37°C, 22°C, and 4°C to late logarithmic phase. Three types of OMVs preparation were used depending on experiment objectives and requirements: crude OMVs, >100 kDa OMVs, and density gradient purified OMVs as indicated during analysis of results and their discussion. The crude OMV preparation contained the whole population of OMVs. It was used for determination of concentration and size distribution as well as for analysis of OMVs interactions with complement components *in vitro*. Due to temperature‐dependent expression, the risk of fimbrial and flagellar contamination in 37°C OMVs preparations was low (Maclagan and Old 1980; Minnich and Rohde 2007; Skurnik 1984). This was confirmed by TEM (Figure S1C). However, contamination with other bacteria/medium derived components could not be excluded. The >100 kDa OMVs preparation was obtained after OMVs concentration with the use of 100 kDa cut‐off membrane. The higher purity allowed for its usage in *in vivo* studies. Finally, for chemical analysis of OMVs‐associated LPS the density gradient purified OMVs were used. The bacterial origin of vesicles was confirmed by the detection of ECA with the use of specific mAbs (Figure S2). Moreover, isolated YeS‐c OMVs were bioactive since they potently inhibited lytic activity of the YeO3‐specific bacteriophage φYeO3‐12 (Figure S3). The ability of OMVs secreted by bacteria grown at 4°C, 22°C and 37°C to neutralize phage was higher when compared with YeS‐c_37°C LPS. The highest inhibitory potential was found for OMVs derived from bacteria cultivated at 4°C.

### 3.1 LPS Chemotype and Growth Conditions Affect *Y. enterocolitica* O:3 OMV Secretion and Their Size

The wide range of growth temperatures and unique arrangement of the YeO3 LPS structure (including S, Ra, Rd1, Re chemotypes) were expected to be reflected by differences in OMVs secretion capability, size and concentration. To verify that, the dimensions and concentrations of OMVs were determined using the NTA. As mentioned above, to avoid OMV selection resulting from concentration and further purification steps, crude OMVs preparations were used.

To determine the influence of LPS chemotype on OMVs release, YeS‐c, YeRa‐c, YeRd1‐c and YeRe‐c bacteria were cultivated at 37°C. The OMV yields among analyzed chemotypes differed significantly (Kruskal‐Wallis ANOVA: *p* < 0.0001), with the highest amount of OMVs secreted by Rd1 chemotype bacteria with LPS limited to the lipid A and IC oligosaccharide (lipid A‐IC) (Table 3). Those bacteria produced 3 to 6‐fold more OMVs than bacteria with LPS of other chemotypes with statistically significant difference in comparison with the YeRe‐c strain (Dunn's post‐hoc test*: p* = 0.0065). Moreover, growth temperature also affected OMVs release. The amount of OMVs secreted by YeS‐c bacteria propagated at 37°C, 22°C and 4°C differed markedly (Kruskal‐Wallis ANOVA: *p* = 0.014) with a significant difference between bacteria cultivated at 22°C and 37°C (Dunn's post‐hoc test*: p* = 0.049). Moreover, the expression of virulence plasmid (strain YeO3‐Ail) was also associated with two times higher OMV secretion when compared with the corresponding pYV‐cured variant (although only two OMVs isolations for each strain were performed).

**TABLE 3:** The influence of LPS chemotype, growth temperature and pYV expression on *Y. enterocolitica* O:3 OMV secretion level and OMV size.

Growth temperature	YeO3 strain	n^1^	OMV concentration(×10^10^ particles/ml)		OMVs size (nm)			
			Mean	Range	Mean	Range	Mode	Range
4°C	(YeS‐c)^4^	3	6.5	3.0–8.8	211	176–248	143	103–187
22°C^5^		3	5.1	4.1–6.0	189	181–189	139	115–170
37°C^5^		5	**9.3**	**8.3**–**9.3**	183	145–236	127	94–155
37°C^2^	YeRa‐c	3	5.9	5.0–5.8	172	170–176	116	108–125
	YeRd1‐c^3^	4	**33.5**	**17.9**–**69.0**	160	128–233	107	87–147
	YeRe‐c^3^	3	5.0	4.7–5.3	158	147–168	124	118–133
	YeO3‐Ail	2	26.6	24.4–27.9	146	137–155	106	100–113
	YeO3‐c‐Ail	2	12.3	8.06–16.6	157	153–161	124	122–127

^1^number of biological repeats.

^2^significant differences among amounts of OMV released by bacteria of various chemotypes (Kruskal‐Wallis ANOVA: *p* < 0.0001).

^3^significant difference between amounts of OMV released by bacteria of Rd1 and Re chemotypes (Dunn's post‐hoc test: *p* = 0.0065).

^4^significant differences among amounts of OMV released by bacteria of S chemotype, cultivated at various temperatures (Kruskal‐Wallis ANOVA: *p* < 0.014).

^5^significant difference between amounts of OMV released by bacteria of S chemotype, cultivated at 22°C and 37°C (Dunn's post‐hoc test: *p* = 0.049).

When analysing the OMVs size (whole population) of bacteria grown at 37°C, the mean diameter of crude OMVs secreted by bacteria synthesising LPS truncated to lipid A‐Kdo (YeRe‐c_37°C) or to lipid A‐IC (YeRd1‐c_37°C) was smaller by approximately 20 nm in comparison with OMVs of YeS‐c_37°C (160 nm *vs*. 180 nm, Table 3), but this difference was not statistically significant (Kruskal‐Wallis one‐way ANOVA *p* = 0.593). The growth temperature seemed to affect the size of YeS‐c OMVs more clearly. The average vesicle diameter was the lowest when parental strain was cultivated at 37°C, medium at 22°C, and the highest at 4°C, with approximately 30 nm difference between bacteria grown at temperature extremes (183 nm *vs*. 211 nm, Table 3). However, this was also not statistically significant (Kruskal‐Wallis one‐way ANOVA *p* = 0.582). The higher proportion of the largest OMVs (≥150 nm) budding off or on the surfaces of rod‐shaped bacterial cells grown at 4°C was also confirmed in SEM analysis (Figure S1A, B). Interestingly, for bacteria cultivated at 4°C, OMV aggregations were observed frequently around the sites of cell splicing (Figure S1A).

**FIGURE 2:**
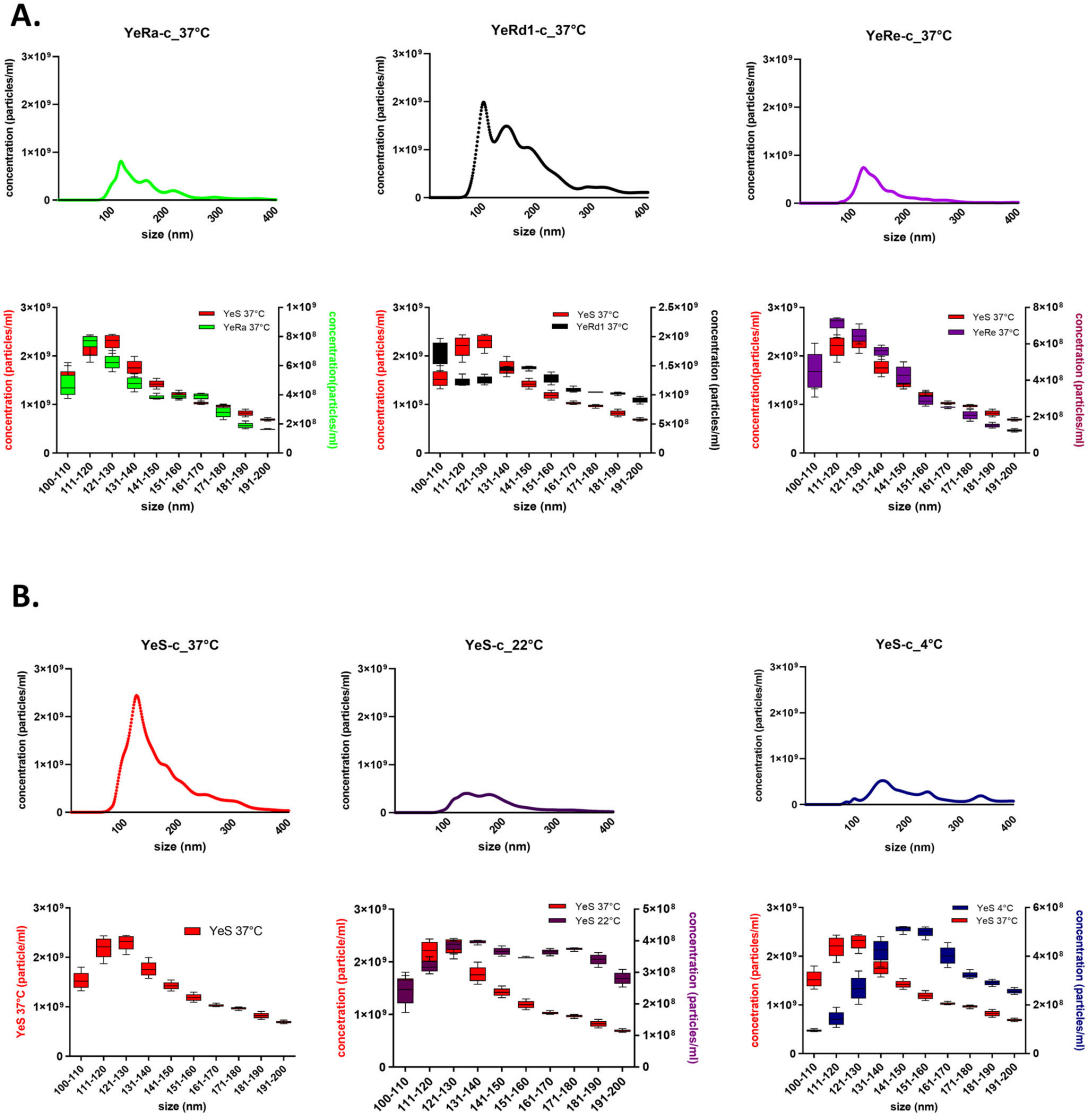
The influence of LPS chemotype and growth temperature on pYV‐cured *Yersinia enterocolitica* O:3 OMV secretion (size ≤ 400 nm) and size distribution (size range 100–200 nm) quantified by NTA. (A) The influence of LPS chemotype on YeO3‐c OMV secretion and size distribution. The YeRa‐c_37°C (green), YeRd1‐c_37°C (black) and YeRe‐c_37°C (purple) OMVs size profiles were compared to that of YeS‐c_37°C (red) OMVs; (B) The influence of growth temperature on OMV secretion and size distribution. The YeS‐c_4°C (blue), and YeS‐c_22°C (purple) OMV size profiles were compared to that of YeS‐c_37°C (red) OMVs. The results for each strain are presented as mean values of at least three biological repeats.

Differences in OMVs dimension among analyzed strains were even more evident when the analysis was restricted to the 100–199 nm size range. Independently of LPS chemotype a significantly higher proportion of 100–149 nm fraction than of 150–199 nm for YeS‐c_37°C (66%), YeRa‐c_37°C (66%), YeRd1‐c_37°C (57%), YeRe‐c_37°C (74%) (Figure 2A, B, Table S1) was observed. The YeRd1‐c_37°C differed from other strains due to higher (20%) proportion of OMVs sized 50–99 nm than 100–199 nm when compared with 11% for YeS‐c, 8% for YeRa‐c and 7% for YeRe‐c.

The growth temperature appeared to be the main player affecting distribution of OMV size (Figure 2B). Confirming the SEM analysis (Figure S1B), in the size range 100–199 nm lowest temperature (4°C) was generally associated with the production of larger vesicles. In contrast to YeS‐c_37°C, the majority (56%) of OMVs released by YeS‐c propagated at 4°C had the diameter ≥150 nm and in the range of 100–300 nm it increased to >70%, whereas for the same strain grown at 22°C and 37°C it equalled 47% and 62%, respectively. In ranges of 100–149 nm and 150–199 nm, YeS‐c_22°C bacteria produced an evenly distributed population of OMVs (50%/50%).

These results indicate that both LPS chemotype and growth temperature affect the OMVs secretion and their size. The reduction of the LPS molecule to lipid A‐IC stimulates OMVs release, whereas cultivation of bacteria at 4°C appears associated with a production of the largest vesicles.

### 3.2 The *Y. enterocolitica* O:3 OMV‐Derived LPSs Reflect Structures of Cell‐Derived LPSs

Both polysaccharide LPS region as well as lipid A acylation pattern were previously shown to affect OMVs secretion (Elhenawy et al. 2016; Kulp et al. 2015). To analyze Ye OMV‐associated lipid A composition of YeS‐c bacteria cultivated at 37°C, 22°C, 4°C and YeRa‐c, YeRd1‐c and YeRe‐c propagated at 37°C, density gradient purified OMVs and cell‐derived LPS were hydrolyzed and water‐insoluble sediments (including lipid A) and soluble supernatants containing IC‐OPS and/or IC‐OC or IC were isolated. The supernatant was fractionated by size‐exclusion chromatography (Figure S4) and obtained fractions were analyzed by MALDI‐TOF MS or NMR spectroscopy.

#### 3.2.1 OMV‐ and Cell‐Derived OPS Structures are Identical

Chromatograms of poly‐ and oligosaccharides isolated from hydrolyzed cell‐derived LPSs indicated fractions of OPS (12–17 min) for YeS‐c_37°C (Figure S4A), YeS‐c_22°C (Figure S4B), and YeS‐c_4°C (Figure S4C). Fractionation of soluble supernatants obtained from hydrolyzed OMV allowed for OPS identification (Figure S4D, E, F), although they showed weaker intensity compared to other non‐sugar components of OMV (18–20 min and 24–33 min). OPS fractions isolated from YeS‐c OMV‐derived LPSs and cell‐derived LPSs were analyzed by ^1^H NMR spectroscopy (Figure S5) yielding similar spectra with signals of protons H1‐H6 attributed to the homopolymer of →2)‐β‐l‐6dAlt in accordance with published data (Hoffman et al. 1980; Muszyński et al. 2013). Screening for OC and IC oligosaccharides was performed by MALDI‐TOF MS for each 1 mL fraction, however ions attributed to core oligosaccharides were not identified as suppressed by other constituents of OMVs.

#### 3.2.2 OMV‐Derived Lipids A Reflect Structures of Cell‐Derived Lipids A

**FIGURE 3:**
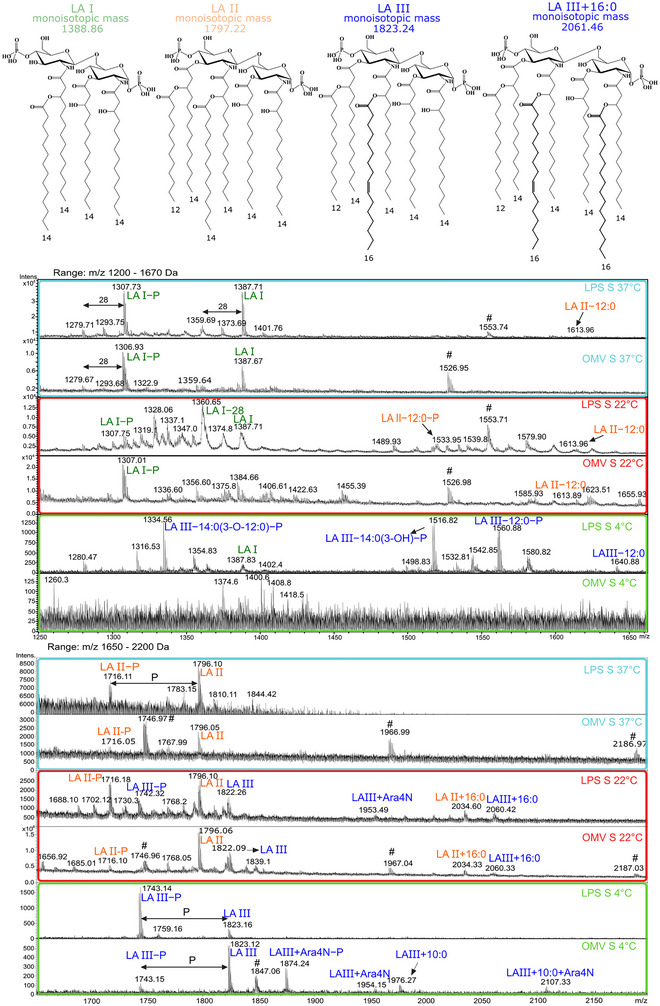
MALDI‐TOF mass spectra of cell‐derived lipids A and OMV‐derived lipids A of *Y. enterocolitica* O:3 isolated from YeO3 cultures of chemotype S at 37°C (bright blue frame), 22°C (red frame), and 4°C (bright green frame). The panels are described in the upper right corner of the spectrum. The spectra are presented in two ranges: 1200–1670 Da (upper panel) and 1650–2200 Da (lower panel). Interpretation based on published structures of *Y. enterocolitica* lipids A (Aussel et al. 2000; Guo et al. 1998; Oertelt et al. 2001; Pérez‐Gutiérrez et al. 2010; Reinés et al. 2012a). LA, lipid A; LA I, II, III, forms of YeO3 lipids A as indicated by chemical structures on top and coloured in green, orange, and blue, respectively; Ara4N – L‐α‐aminoarabinose, 16:0 – hexadecanoic acid (palmitic acid); 14:0 – tetradecanoic acid, 14:0(3‐OH) ‐ 3‐hydroxytetradecanoic acid; 12:0 – dodecanoic acid, P, phosphate group. Characteristic acyl residues in structures are marked in bold.

To compare lipid A composition, fractions from cell‐derived LPS and OMV‐derived LPS were analyzed by MALDI‐TOF MS (Figure 3 and Figure S6). Analyses were limited to the comparison of MS of YeO3 lipids A and interpretation of negatively charged ions (Table S2) based on previously published structures mainly of *Y. enterocolitica* O:8 lipids A (Guo et al. 1998; Oertelt et al. 2001; Pérez‐Gutiérrez et al. 2010; Reinés et al. 2012a), including YeO3 lipids A from bacteria cultivated at 37°C and 21°C (Aussel et al. 2000, Fernandez‐Carrasco et al. 2025).

Figure 3 shows comparison of mass spectra recorded for lipids A characteristic for cell‐ and OMV‐derived YeO3 LPSs. In general, all major forms previously described for *Y. enterocolitica*, including YeO3, were identified and referred herein as LA I, LA II, and LA III (Figure 3, inset chemical structures). Most of observed ions were explained in Table S2 indicating the exact position of acyl chains at position 2, 3, 2’, and 3’ of the carbohydrate backbone. The LA I is bis‐phosphorylated tetraacylated LA substituted with 14:0(3‐OH), 14:0(3‐OH), and 14:0(3‐O‐14:0) fatty acids at positions 2, 3, and 2’, respectively. LA II is bis‐phosphorylated and hexaacylated form, substituted with 14:0(3‐OH), 14:0(3‐OH), 14:0(3‐O‐14:0), and 14:0(3‐O‐12:0) at positions 2, 3, 2’, and 3’, respectively. LA III is hexaacylated form that differs from LA II by the presence of 16:1 secondary acyl chain at position 2’ instead of 14:0 (Figure 3, inset chemical structures).

For YeS‐c_37°C, identical forms of lipids A were observed for cell‐ and OMV‐derived LPSs such as bis‐ and monophosphorylated tetraacylated LA I and bis‐phosphorylated and hexaacylated LA II (Figure 3). The most abundant negatively charged [M−H]^−^ ions were attributed to bis‐ and monophosphorylated tetraacylated LA I (*m/z* 1387.71) and LA I−P (*m/z* 1307.73) (Table S2) with lower intensity ions attributed to bis‐ and monophosphorylated hexaacylated LA II (*m/z* 1796.10) and LA II−P (*m/z* 1716.11), respectively.

Similarly, for YeS‐c_22°C, identical forms of lipids A were observed for cell‐ and OMV‐derived LPSs (Figure 3, Table 4), however a higher deacylation level of LA II, represented by LA II−12:0 (m/z 1613.96) and a higher level of dephosphorylation to monophosphorylated forms were observed for cell‐derived LPS and resulted from phenol/water extraction of LPS.

**TABLE 4:** Comparison of lipid A forms identified by MALDI‐TOF MS analyses in cell‐derived LPS and OMV‐derived LPS isolated from pYV‐cured *Y. enterocolitica* O:3 S (cultured at 37°C, 22°C, 4°C), Ra, Rd1, and Re (cultured at 37°C).^1^

Lipid A form/ chemotype/ growth temperature/	YeS						YeRa		YeRd1		YeRe	
	4°C		22°C		37°C		37°C					
	LPS	OMV	LPS	OMV	LPS	OMV	LPS	OMV	LPS	OMV	LPS	OMV
**LA I** (tetraacylated) *m/z* 1387.71	•	*	••	••	•••	•••	•••	•••	•••	•••	•••	•••
**LA II** (hexaacylated) *m/z* 1796.10	**—**	**—**	••	•••	•	•	••	••	••	••	••	**—**
**LA II +P** (hexaacylated) *m/z* 1876.7	**—**	**—**	**—**	**—**	**—**	**—**	**—**	•	*	•	**—**	**—**
**LA III** (hexaacylated) *m/z* 1822.1	**•••**	**•••**	**••**	**••**	**—**	*	**—**	•	**—**	•	*	**—**
**LA III+Ara4N** (hexaacylated) *m/z* 1953.49	**—**	**—**	**—**	**•**	**—**	**—**	*	**—**	**—**	**—**	**—**	**—**
**LA II+16:0** (heptaacylated) *m/z* 2034.60	**—**	**—**	**•**	**•**	**—**	**—**	**—**	**—**	**—**	**—**	**—**	**—**
**LA III+16:0** (heptaacylated) *m/z* 2060.33	**—**	**—**	•	•	**—**	**—**	**—**	**—**	**—**	**—**	**—**	**—**
**LA III+10:0** (heptaacylated) *m/z* 1976.3	**—**	•	**—**	**—**	**—**	**—**	**—**	**—**	**—**	**—**	**—**	**—**
**LA III+10:0+Ara4N** (heptaacylated) *m/z* 2107.3	**—**	•	**—**	**—**	**—**	**—**	**—**	**—**	**—**	**—**	**—**	**—**

^1^LA, lipid A; LA I, II, III, forms of YeO3 lipids A as shown in Figure 3 and explained in Table S1; LA III contains 16:1Δ^9^ – *cis*‐hexadec‐9‐enoic acid (16:1) as a secondary linked acyl at position 2’. Ara4N‐L‐α‐aminoarabinose, 16:0 – hexadecanoic acid (palmitic acid); 10:0 – decanoic acid; 14:0(3‐OH) ‐3‐hydroxytetradecanoic acid; P, phosphate group.

Number of •symbols indicate relative intensity of ions in the spectrum.

*trace ion.

Differences in *m/z* values of 28 and 26 Da indicated heterogeneity of LA I and LA II in YeS‐c_37°C and YeS‐c_22°C LPS and OMVs attributed to substitution pattern characterized by shorter acyl residues. The major difference between 37°C and 22°C cultures was the appearance of hexaacylated LA III forms (LA III—ion at *m/z* 1822.26, LA III+Ara4N—ion at *m/z* 1953.49) and heptaacylated LA II and LA III forms (LA II+16:0 – ion at *m/z* 2034.60, LA III+16:0– ion at *m/z* 2060.42) (Table 4 and Table S2). However, LA III+Ara4N (the ion at *m/z* 1953.49) was not observed for OMV‐derived LPS (Figure 3).

Original data were for the first time provided for Ye lipid A derived from the culture at 4°C. For YeS‐c_4°C, mainly LA III forms of lipids A were observed for cell‐ and OMV‐derived LPS (Figure 3), however higher heterogeneity and lower level of deacylation was observed for OMV‐derived LPS. For both lipid A sources, no deacylated LA II forms were found and only trace amounts of tetraacylated LA I. The hexaacylated LA III prevailed in both lipid A samples (LA III and LA III+Ara4N) (ions at *m/z* 1823.12, 1954.15).

Finally, structures of cell‐ and OMV‐derived lipids A of YeRa‐c_37°C, YeRd1‐c_37°C, YeRe‐c_37°C were compared. Analysis of MALDI‐TOF mass spectra revealed no differences between lipids A present in YeO:3 S and Re strains (Figure S6). The LA III form was observed for OMV‐derived S, Ra, and Rd1 LPS only. For Ra and Rd1 mutants, lower intensity ions attributed to LA II+P (the presence of PP group) were observed both for OMV‐ and cell‐derived lipids A. Results of this comparative analysis are summarized in Table 4.

Our findings indicate no difference in cell‐ and OMVs‐associated LPS structure. The growth temperature affects lipid A acylation pattern, with decreased acylation at 37°C. The hexaacylated and 16:1 substituted LA III form was predominant when bacteria were cultivated at 4°C, tetraacylated LA I, hexaacylated LA II and LA III coexisted at 22°C, whereas tetraacylated LA I was predominant at 37°C.

### 3.3 The *Y. enterocolitica* O:3 OMV Surface‐Expressed LPS and YadA Affect Bactericidal Activity of Human Serum

Culture supernatants of cured YeO3 (representing chemotypes S, Ra, Rd1, Re) as well as pYV+ YeS variants were used as a source of OMVs for screening of the LPS chemotype‐dependent efficiency of OMVs to modulate the bactericidal activity of NHS against the complement‐sensitive YeS‐c strain grown at 37°C.

#### 3.3.1 Long Chain OPS Appears to Play a Crucial Role in Complement Consumption by OMVs and Decrease of Bactericidal Activity of Serum

**FIGURE 4:**
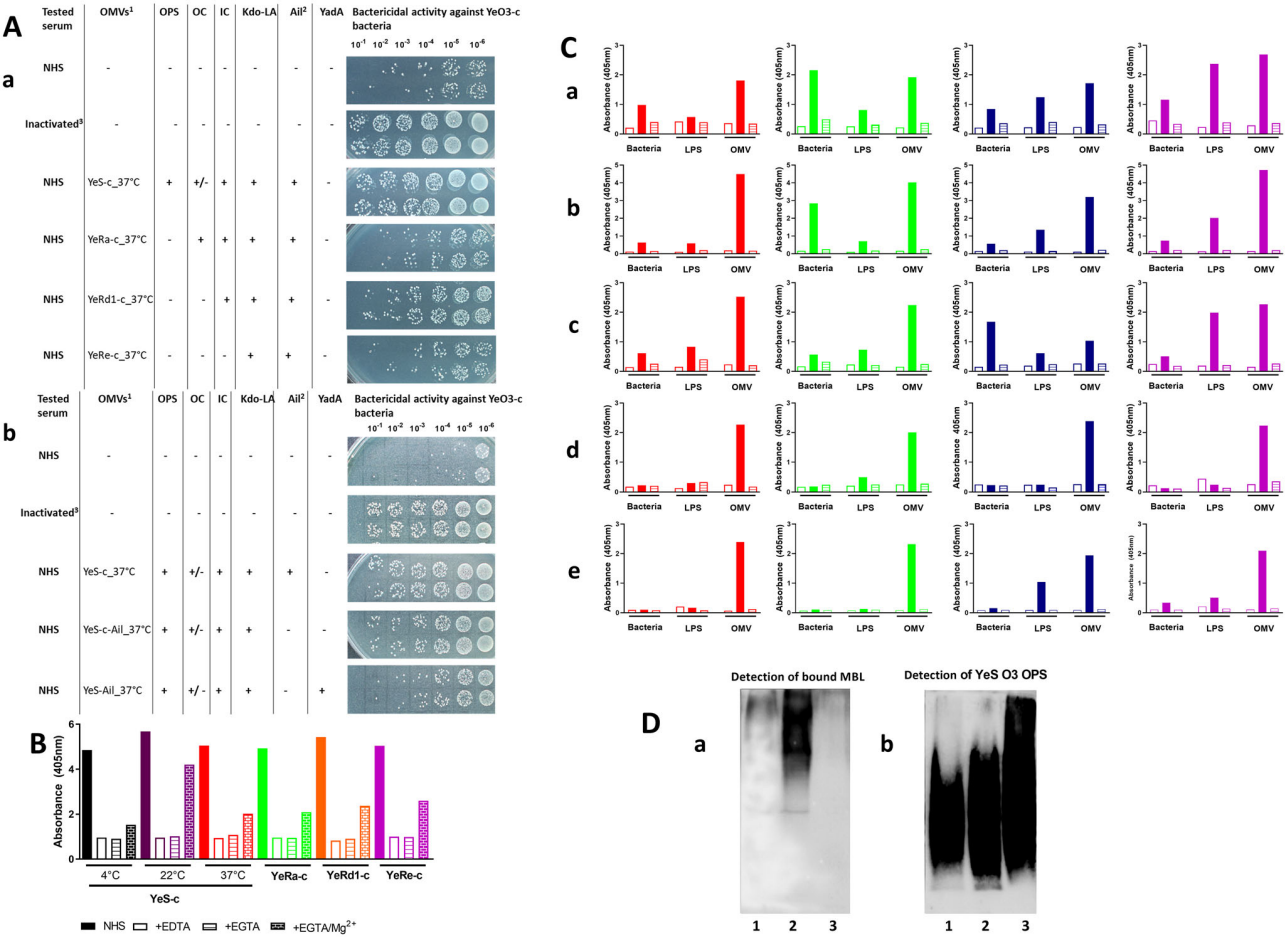
(A) The impact of OMVs on germicidal activity of serum against YeO3‐c bacteria. The influence of OMVs‐associated with: LPS polysaccharide chain length (a) and pYV‐coded factors (b). Data from one of at least two experiments with the use of separately prepared culture supernatants with similar results are presented. ^1^sterile cell culture supernatants (secreted by bacteria grown to OD_600_ = 0.6) were used as a source of OMVs; ^2^expression at 37°C only; ^3^NHS inactivated 30 min at 56°C. (B) *Y. enterocolitica* O:3 OMV‐induced complement activation in the presence of calcium and magnesium chelators. ELISA plates were coated with 10^8^ of OMVs secreted by YeS‐c bacteria grown at 4°C, 22°C and 37°C and with OMVs of YeRa‐c, YeRd1‐c and YeRe‐c variants propagated at 37°C. After incubation with/without EDTA, EGTA or EGTA/Mg^2+^ (EGTA supplemented with Mg^2+^ ions), products of C3 activation were detected with specific antibodies. Data from one of two experiments with similar results are presented. (C) Comparison of complement activation and MBL binding by *Yersinia enterocolitica* O:3 bacterial cells, LPS and OMVs. ELISA plates were coated with 50 ng/well of bacteria, LPS or OMVs. The deposition of C3 (a, possible AP, CP and LP involvement), C4 (b, possible CP and LP involvement) or C4 LP‐dependent (d) activation products, TCC formation (c, possible AP, CP and LP involvement) and MBL‐binding (e) was analyzed after preincubation with normal human serum (filled columns) or with EDTA‐treated NHS (stripped columns) or without serum (open columns, negative control). Data from one of two experiments with similar results are presented. Dots represent individual OD values for each well. (D) Recognition of *Y. enterocolitica* O:3 OMVs by human mannose‐binding lectin. YeS‐c_37°C bacteria (1), OMVs (2) and LPS (3) were separated in SDS‐PAGE. After transfer to nitrocellulose and incubation with NHS, the bound MBL was detected with specific mAb (a). The experiment was performed at least 5 times. To control the loading of bacteria, LPS or OMVs the presence of OPS in separated samples was confirmed with anti‐6‐deoxy‐L‐altropyranose mAbs (b).

Preincubation of NHS with sterile spent media (of bacteria grown at 37°C and representing various LPS chemotypes) was associated with attenuated bactericidal activity against YeS‐c indicator bacteria. The OMVs of S‐type bacteria reduced the germicidal potency more efficiently than OMVs secreted by bacteria with truncated OPS of LPS (Figure 4A, a).

#### 3.3.2 The Expression of Virulence Plasmid‐Encoded Virulence Factors Decreases Complement Consumption by OMVs

Next, the influence of OMV surface‐expressed serum‐resistance factor inhibiting complement activation *via* factor H and C4bp binding was tested. The effect of YeO3 variants with expression of YadA (without Ail, pYV+, YeS‐Ail), with expression of Ail (without YadA, pYV‐, YeS‐c) and without expression of both YadA and Ail (pYV‐, YeS‐c‐Ail) was compared.

The results indicated lower complement‐consumption ability for OMVs secreted by pYV‐positive bacteria (YeO3‐Ail) in comparison with vesicles released by pYV‐cured bacteria without the expression of serum‐resistance factors) (Figure 4A, b).

Altogether, the above data indicate that OMVs secreted by YeO3 affect the germicidal potential of NHS. The vesicles secreted by pYV‐cured bacteria markedly reduced lytic activity of NHS against the indicator bacteria, whereas those produced by pYV‐positive bacteria (able to bind complement regulators) had a weaker effect.

### 3.4 *Y. enterocolitica* O:3 OMVs Activate Alternative, Classical and Lectin Pathways of Complement

To analyse ability of OMVs to activate complement, crude OMVs were used for microtiter plate coating. Complement activation was indicated by detection of complement C3 or C4 activation products as well as TCC formation.

In preliminary experiments, we tested OMV‐induced complement activation in the presence of calcium or magnesium chelators. The pre‐incubation of NHS with EDTA (with high affinity to both Ca^2+^ and Mg^2+^ ions) and EGTA (with high affinity to Ca^2+^ and low to Mg^2+^ ions) diminished the C3 activation level (Figure 4B). Supplementation of EGTA‐treated serum with magnesium cations (essential for alternative pathway) caused only a partial recovery of complement activation suggesting some involvement of classical and/or lectin pathways.

Next, we demonstrated that independently of LPS chemotype, OMVs were able to activate complement C3 (Figure 4C,a) and C4 (Figure 4C, b) leading to TCC formation (Figure 4C, c) at a higher level when compared with parental bacterial cells or isolated LPS (Figure 4). We ruled out that observed differences resulted from lower coating efficiency of bacterial cells by detection of ECA on the surface of both bacteria and OMVs (Figure S2). The OMVs‐induced C4 activation may proceed with the involvement of either classical or lectin pathway (Figure 4C, b, d). The application of CP exclusionary conditions (Petersen et al. 2001), revealed that independently of LPS chemotype, OMVs can activate complement *via* the LP (Figure 4C, d). Moreover, our data indicated that human serum mannose‐binding lectin (MBL) interacts with OMVs and induces complement activation (Figure 4C, d, e). Trace or no binding of other complement‐activating lectins (ficolins‐1, ‐2 or ‐3) to OMVs was observed (Figure S7). Trace binding of ficolin‐3 to YeS‐c_37°C OMVs may result from its higher concentration in serum in comparison with MBL, ficolin‐1 or ficolin‐2 and possible interaction with natural antibodies reported by Panda et al. (2013) and Man‐Kupisinska et al. (2018). Furthermore, both murine MBLs (MBL‐A and MBL‐C) were found to recognize OMVs (Figure S8).

These results indicate that OMVs secreted by pYV‐negative YeO3 (independently of LPS chemotype), are potent inducers of complement activation. It may proceed through all three canonical routes and leads to TCC formation. MBL interacts with Ye OMVs and that may result in C’ activation *via* the lectin pathway.

### 3.5 High Molecular Weight Fraction of *Y. enterocolitica* O:3 OMVs is Involved in MBL Binding

The OMVs structure responsible for MBL binding was identified in Western blot. First, crude YeS‐c_37°C OMVs, bacteria and LPS were separated in SDS‐PAGE (under reducing condition) and transferred to nitrocellulose membrane. After incubation with high‐MBL NHS, the bound protein was detected with specific monoclonal antibody (Figure 4D). It was demonstrated that MBL recognizes the crude OMVs fractions that migrate slowly in SDS‐PAGE. Trace MBL binding to bacteria or isolated YeS LPS was found in the corresponding regions. Slow migrating MBL‐binding fractions were detected both in crude and density gradient‐purified OMVs secreted by pYV‐negative bacteria of Ra, Rd1 and Re LPS chemotypes (Figure S9A). A similar recognition pattern was found when murine serum was used as the MBL‐A and MBL‐C source (Figure S9B). Contamination with LB medium components was excluded (no reaction with the medium treated according to OMV isolation procedure, Figure S9C). The MBL‐binding structure was thermostable (100°C, 1 h), and resistant to DNase and proteinase K (Figure S9D). Moreover, it was also stable under conditions usually used for LPS poly‐ and oligosaccharide detachment from lipid A: heating of membrane‐immobilized LPS for 1 h at 100°C in 1% acetic acid did not diminish MBL binding, although detection of lipid A with specific antibodies was possible (Figure S9E).

These results indicate that OMVs (independently of LPS chemotype) are decorated with glycoconjugate(s) recognized by human MBL as well as murine MBL‐A and MBL‐C. This entity probably contains a lipid carrier and is distinct from LPS.

### 3.6 *Y. enterocolitica* O:3 OMV‐Induced Complement Activation May Contribute to SIRS Development

**FIGURE 5:**
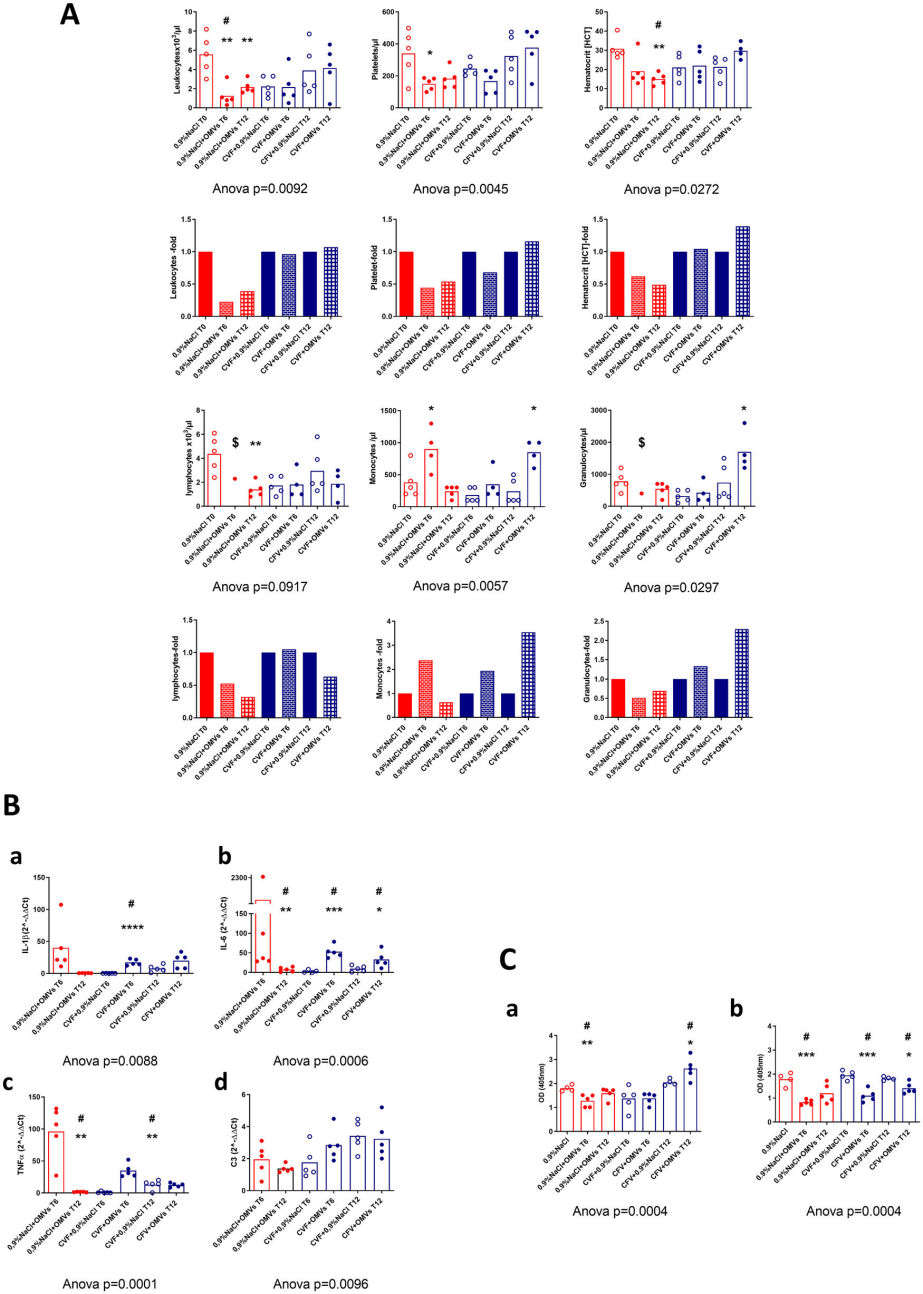
(A) Time‐dependent changes in blood cell counts and haematocrit due to *i.p*. administration of YeS‐c_37°C OMVs to C57Bl mice without (red) and with (blue) CVF‐induced decomplementation (5 animals/group). Data presented as means of absolute numbers (upper graphs) or fold change (lower graphs) to PBS‐treated mice or PBS‐treated decomplemented mice. ^$^in OMVs‐treated normocomplementic mice at T6, the results were obtained for one animal only. Each dot/circle represents an individual mouse. (B) Time‐dependent expression of murine genes coding pro‐inflammatory cytokines [IL‐1β (a), IL‐6 (b), TNF‐α (c)] and complement C3 component (d) due to administration of YeS‐c_37°C OMVs. Red—normocomplementic mice; blue—mice pre‐treated with cobra venom factor. Dots/circles represent individual values for each mouse. (C) The effect of *Yersinia enterocolitica* O:3 OMV administration on the levels on murine MBLs. The MBL‐A (a) and MBL‐C (b) were detected in sera after intraperitoneal administration of YeS‐c_37°C to normocomplementic (red) and decomplemented (blue) mice. Each dot/circle represents an individual mouse. For MBL‐A and MBL‐C detection sera were diluted 500× and 2000×, respectively. The experiment was only conducted once for ethical reasons, but it was preceded by pilot studies to select the optimal time points and the dosage of administered OMVs. For comparison of all analyzed groups Kruskal‐Wallis one‐way ANOVA was used with statistically significant differences against 0.9% NaCl T0 marked with slash mark if *p* < 0.05 after Dunn's post hoc test. For comparison of single tested group to respective control group (0.9% NaCl, CFV+0.9% NaCl T6 or CFV+0.9% NaCl T12 Mann‐Whitney *U* test was used with significant differences marked with asterisk if *p* < 0.05. Open circles/columns—mice treated with PBS, closed circles/columns—mice treated with OMVs.

To test *in vivo* consequences of OMV‐induced complement activation, male C57BL/6 mice were injected intraperitoneally with a non‐lethal dose (15 µg) of YeS‐c_37°C >100 kDa OMVs preparation. Despite lacking the pYV‐associated factors, changes in blood cell counts (CBC) were observed: six and even twelve hours (T6 and T12, respectively) after OMV injections, marked decreases in leukocyte counts (OMV‐treated mice at T6 and T12 *vs*. control mice: *p* = 0.016 in both instances), lymphocyte counts (OMV‐treated mice at T12 *vs*. control mice: *p* = 0.016) and haematocrit (OMV‐treated mice at T6 and T12 *vs*. control mice: *p* = 0.0079 in both instances) were observed (Figure 5A).

To gain insight into the role of complement in OMV‐associated changes in CBC, complement consumption was induced by treating the mice with cobra venom factor (CVF). The effectiveness of CVF administration was verified in Western blot by immunostaining with anti‐C3 antibodies. In all CVF‐treated mice no native C3 α‐chains (band corresponding to 115 kDa) were detected (Figure S10).

The CVF treatment of mice affected their CBC, though not significantly (Figure 5A). Despite this, *i.p*. injection of complement‐depleted mice with OMVs was not associated with a further decrease of leukocytes, platelets, lymphocytes or haematocrit, unlike the changes observed in OMV‐treated mice with an active complement system (in all cases *p* > 0.05, when compared with mice treated with CVF only).

The changes in blood cell counts due to OMV administrations were accompanied by increased expression of pro‐inflammatory cytokine genes in the liver (Figure 5B). Injection of OMVs to normal mice resulted in the induction of IL‐1β‐, IL‐6‐ and TNFα‐specific mRNA at T6, followed by their decreased expression 6 h later (T12). A similar pattern of response to OMVs was found in complement‐depleted mice, but the expression of these genes was much weaker. The OMV‐induced changes in cytokine expression in naive mice were 2.3–9.7 times higher (however the differences were not significant statistically) than in decomplemented mice (mean 2^−∆∆Ct^ values for IL‐1: 40.13 *vs*. 17.31; IL‐6: 514.7 *vs*. 52.94 and TNF‐α: 96.24 *vs*. 34.95, respectively). Remarkably, CFV treatment increased C3 gene expression over time compared with animals treated with 0.9% NaCl (mean 2^−∆∆Ct^ values at T6: 1.768 and at T12: 3.414) and further increased expression at T6 after OMVs treatment (mean 2^−∆∆Ct^ = 2.848).

In mice with the intact complement system, the YeS‐c_37°C OMV injections were associated with significantly reduced levels of MBLs, MBL‐A (*p* = 0.019) and MBL‐C (*p* = 0.016) at T6 when compared with their 0.9% NaCl‐treated counterparts (Figure 5C). Complement depletion with CVF did not markedly influence MBL‐A/‐C concentrations when compared with control (0.9% NaCl‐treated) mice (*p* = 0.111 and *p* = 0.556, respectively). However, treatment of complement‐depleted mice with YeS‐c_37°C OMVs led to a significant decrease of levels of MBL‐C at T6 (*p* = 0.0079) and T12 (*p* = 0.0317), when compared with corresponding, CVF‐treated reference groups.

The data described above suggest a contribution of OMV‐induced complement activation to the development of the systemic inflammatory response. Regardless of complement status, YeS‐c_37°C OMVs were associated with consumption of MBL‐C.

### 3.7 *Y. enterocolitica* O:3 OMVs Distribution in Mice

**FIGURE 6:**
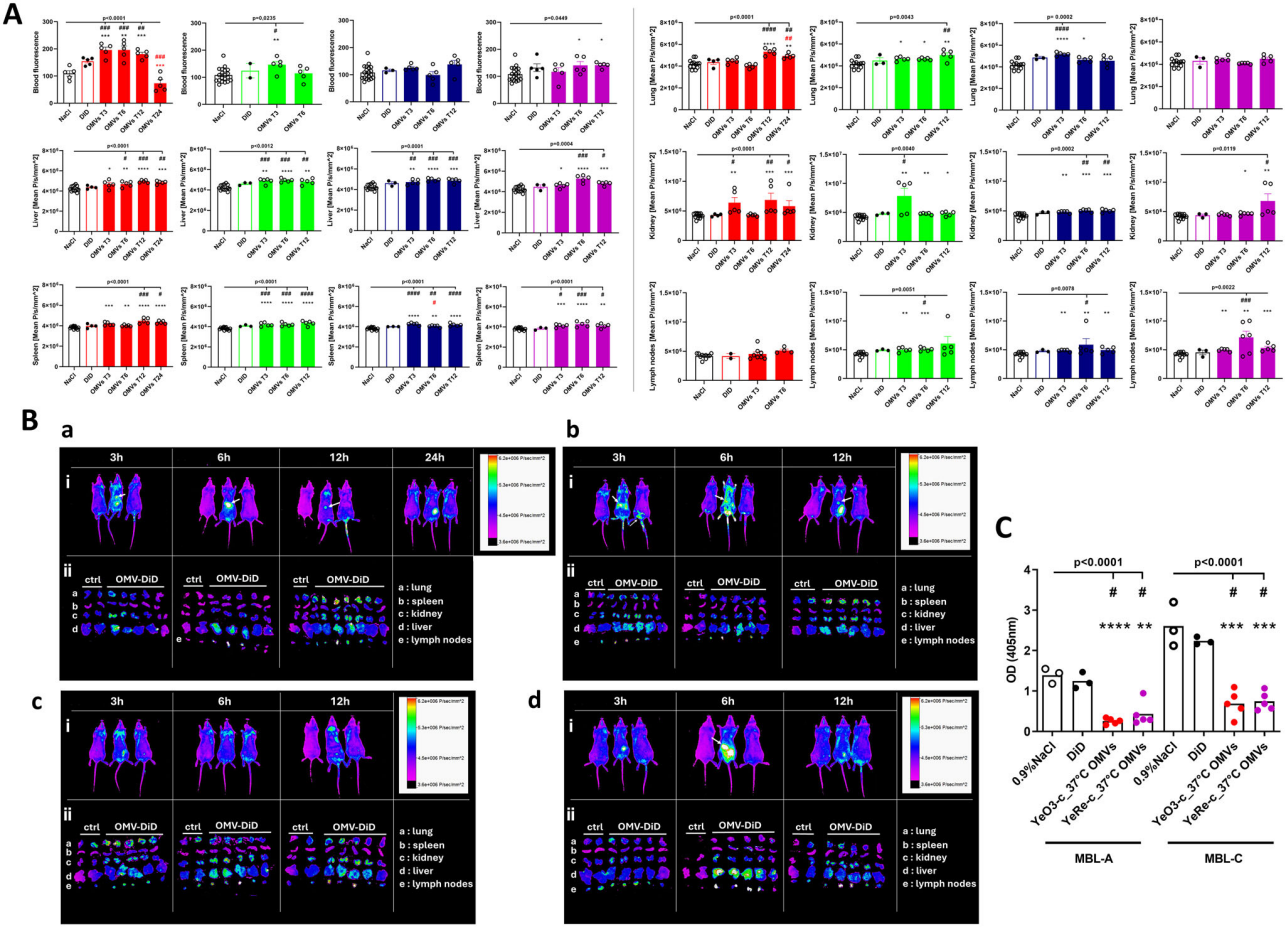
(A) Time‐dependent biodistribution of DiD‐labelled OMVs secreted by *Y.enterocolitica* O:3 bacteria expressing S‐, Ra‐, Rd1‐ and Re‐ LPS chemotypes. Red‐, green‐, blue‐ and purple filled columns present results obtained from animals treated *i.p* with 15 µg of YeS‐c_37°C, YeRa‐c_37°C, YeRd1‐c_37°C and YeRe‐c_37°C DiD‐labelled OMVs, respectively. Red‐, green‐, blue‐ and purple open columns present results from BALB/c mice (5 mice/group) treated with DiD‐NaCl, whereas black open columns—with 0.9% NaCl only. Error bars demonstrate SEM. Each dot/circle represents an individual mouse. Black lines indicate statistically significant differences (*p* < 0.05) in fluorescence intensity among all analyzed groups (Kruskal‐Wallis one‐way ANOVA) and slash marks indicate the results of post‐hoc analysis (Dunn's test) against the 0.9% NaCl group). Asterisk marks indicate the significant difference of analyzed group in comparison with 0.9% NaCl‐treated mice (Mann‐Whitney *U* test). The statistically significant differences to T3 in the Kruskal‐Wallis and Mann‐Whitney *U* tests are indicated with red marks when analysing time‐dependent changes in OMVs‐associated fluorescence. (B) The analysis of the OMV biodistribution kinetics in mice. DiD‐labelled YeS‐c_37°C (a), YeRa‐c_37°C (b), YeRd1‐c_37°C (c) and YeRe‐c_37°C (d) OMVs were administered intraperitoneally. The *in vivo* (i) and *ex vivo* (ii) fluorescence was acquired at indicated time‐points. Panel i: The left mouse was treated with 0.9% NaCl, the middle mouse was treated with DiD‐labelled OMVs, and the right mouse was treated with DiD‐NaCl. Arrows indicate the fluorescence most likely associated with the gallbladder. Panel ii: ctrl—organs of control (0.9% NaCl‐ and DiD‐NaCl‐treated mice); OMVs—organs of mice treated with DiD‐labelled OMVs. The experiment was only conducted once for ethical reasons, but it was preceded by pilot studies to determine the optimal time points and the dosage of administered of OMVs. (C) The influence of *Y. enterocolitica* O:3 OMV administration on the level of MBLs in bile. Gallbladders were isolated at T6 from control mice (*n* = 3) and mice treated with YeS‐c_37°C (*n* = 5) or YeRe‐c_37°C (*n* = 5) OMVs. The changes in MBL‐A and MBL‐C levels in bile (diluted 10 ×) were analyzed in functional ELISA, using yeast mannan as target for MBL. Each dot/circle represents individual mouse. Horizontal lines show significant differences (*p* < 0.05) among analyzed groups (Kruskal‐Wallis one‐way ANOVA); slash marks indicate significant differences to 0.9% NaCl group (Dunn's post‐hoc test); asterisks marks show differences between tested group and 0.9% NaCl group in Mann‐Whitney *U* test.

To test the influence of LPS chemotype on the biodistribution of OMVs, BALB/c mice were injected intraperitoneally with 15 µg of lipophilic fluorescent dye (DiD)‐labelled YeS‐c_37°C, YeRa‐c_37°C, YeRd1‐c_37°C or YeRe‐c_37°C OMVs (crude OMVs). The fluorescence of isolated organs and whole body was tested 3, 6 and 12 h after OMVs injection (Figure 6A, B). Mice treated with YeS‐c_37°C OMVs were monitored also at T24. Since there were no significant differences in the fluorescence among control mice treated with 0.9% NaCl, they were included into a single, common reference group for each tissue analyzed. The fluorescence of tissues among mice treated with DiD‐NaCl was also similar and was higher only in the spleens and lymph nodes when compared with spleens and lymph nodes from mice treated with 0.9% NaCl (Figure S11).

In the blood, fluorescence of OMVs with LPS decorated with OPS chains or OC oligosaccharide were detectable 3 h after *i.p*. injection (Figure 6A, B). A significant difference from 0.9% NaCl‐treated mice at T3 was observed for YeS‐c_37°C (*p* = 0.0079) and for YeRa‐c_37°C (*p* = 0.016). In contrast, the YeRd1‐c_37°C OMV‐associated fluorescence was undetectable in the blood at any time point tested, suggesting rapid clearance of those extracellular vesicles. The YeRa‐c_37°C OMVs were removed from blood at T6 (T12 was not analyzed because no whole blood samples were available). The clearance of OMVs with S‐type LPS was much slower: they were detectable in the blood within 3–12 h (significant differences *vs*. control mice at T6 and at T12 after administration (*p* = 0.0079).

The liver and spleen were the major sites of OMVs accumulation (Figure 6A). Regardless of the LPS chemotype, the fluorescence of the livers from OMV‐treated mice was significantly higher than that of control animals within three hours after the treatment (Figure 6A, control *vs*.: YeS‐c_37°C: *p* = 0.038; YeRa‐c_37°C: *p* = 0.0078; YeRd1_37°C: *p* = 0.036; YeRe‐c_37°C: *p* = 0.018). Furthermore, no significant changes in the fluorescence were observed among mice exposed to OMVs for 3, 6, 12 and even 24 h. A similar pattern of fluorescence detection was found for spleens.

The sequestration of OMVs by organs located behind the peritoneum differed significantly and seemed to be affected by the LPS chemotype. YeRd1‐c_37°C OMVs were rapidly accumulated in lung tissue (Figure 6A). Already 3 h after injection, the fluorescence was significantly higher in comparison with control (*p* = 0.036). After that, the signal decreased and became undetectable at T12. Similarly, the fluorescence associated with YeRa‐c_37°C OMVs was detectable in the lung 3 h after treatment (*p* = 0.038), however, it remained significantly elevated compared with control at T12 (*p* = 0.014). In contrast, the accumulation of YeS‐c_37°C OMVs in lung was delayed, and they were observed only 12–24 h after *i.p*. treatment (*p* = 0.034 in both cases).

YeRd1‐c_37°C, YeRa‐c_37°C, and YeS‐c_37°C OMVs were found in the kidney 3 h after administration (significant differences in comparison with 0.9% NaCl‐treated mice: *p* = 0.0028, *p* = 0.0028 and *p* = 0.0002, respectively) and they were detected even at T12 and T24 (YeS‐c) (Figure 6A). In contrast to other preparations, YeRa‐c_37°C OMVs‐associated fluorescence decreased with time, although the fluorescence at T12 was still markedly higher when compared with control (*p* = 0.025).

The biodistribution pattern observed for OMVs carrying the Re chemotype LPS differed significantly from OMVs of other chemotypes (Figure 6A). They were detected in blood only after 6 h from *i.p*. administration (*p* = 0.0073). Despite being sequestrated in the livers and spleens like other OMVs preparations, they were undetectable in the lungs. In the kidneys, their accumulation seemed to be delayed (at T12) in comparison with extracellular vesicles from bacteria of other chemotypes (at T3 and T12).

Interestingly, OMVs secreted by bacteria with truncated LPS were found to accumulate rapidly in lymph nodes (Figure 6A). The fluorescence was significantly higher in comparison with the reference group within three (for Ra, Rd1 and Re: *p* = 0.036) to twelve (for Rd1 and Re: *p* = 0.027 and *p* = 0.0005, respectively) hours after *i.p*. administration. The sequestration of YeS‐c_37°C OMVs was delayed—it was found at T12 only (*p* = 0.0015).

These results indicate that the OMVs spread rapidly in the murine body after *i.p*. injection. They were transported by the blood and accumulated for at least 12 h, mainly in livers and spleens, independently of the LPS chemotype. Long OPS seemed to protect the OMVs from the clearance from blood, whereas the LPS truncation to core‐lipid A promoted OMVs accumulation in the lymph nodes.

### 3.8 *Y. enterocolitica* O:3 OMV Administration Affects MBL‐A and MBL‐C Levels in the Bile

Analysis of biodistribution of DiD‐labelled OMVs indicated their possible accumulation in the gallbladders (Figure 6B). To test the bile MBL‐OMVs interaction, six hours after YeS‐c_37°C or YeRe‐c_37°C OMVs *i.p*. administration, gallbladders were isolated and MBL‐A and MBL‐C in the bile were determined by ELISA, using yeast mannan as a target for lectins. Treatment with the OMVs was associated with significantly (*p* = 0.036) decreased levels of both forms of murine MBLs in the bile in comparison with control mice (Figure 6C).

These results may indicate that the pathway of the OMV clearance from the murine body leads through the bile into the digestive system, and that is associated with MBL‐A and MBL‐C consumption.

## 4 Discussion

The complement system is an essential arm of innate immunity, contributing to the clearance of infection. However, uncontrolled complement activation may contribute to the clinical manifestation of SIRS/sepsis, often with fatal outcomes. Inhibitors of complement activation have been proposed as therapeutic agents and some of them are already under validation in clinical studies (Mastellos et al. 2019).

OMVs secreted by numerous bacterial species activate complement *in vitro* as previously demonstrated by several research groups (Bjerre et al. 2002; Dehinwal et al. 2021; Pettit and Judd 1992; Tan et al. 2007). Here we used a collection of YeO3 variants, sensitive to bactericidal activity of serum and differing in LPS chemotypes. Since the LPS, the main OMVs envelope component is a potent complement activator, it enabled us to test the ability of YeO3 extracellular vesicles to activate C’ in relation to LPS length. We showed that pre‐incubation of sterile spent media as a source of OMVs with NHS significantly affected its bactericidal activity against indicator bacteria. The outcome depended on the expression of structures that interact with host complement regulators. In the presence of OMVs secreted by serum‐sensitive YeO3‐c bacteria, the bactericidal activity of NHS was decreased markedly while the effect of extracellular vesicles produced by bacteria with YadA expression, able to bind complement inhibitors (FH and C4bp) appeared much weaker. Previously, Dehinwal et al. (2021) demonstrated that OMVs secreted by serum‐resistant wild‐type *Salmonella* as well as its serum‐sensitive mutant (ΔPagC) activated C3. However, only in the case of wild type OMVs it was associated with recruitment of factor H, formation of inactive iC3b and protection of bacteria from serum bactericidal activity. Our data indicate long‐chain LPS as the most potent factor affecting germicidal activity of serum. It was unlikely to be due to consumption of YeO3 OPS‐specific antibodies by OMVs since the level of natural YeO3 antibodies in the serum used was very low (Figure S12). Moreover, enhanced survival of YeO3 bacteria was observed, when serum was preincubated with OMVs secreted by smooth *Escherichia coli* O:16 or *Klebsiella pneumoniae* (Figure S13), supporting the findings of Tan et al. (2007) of complement consuming activity of OMVs surface structures of both parental and other bacterial species. However, it must be noted, that the effects mentioned could be caused by other surface antigens in addition to LPS (Bjerre et al. 2002).

Our results also indicate that YeO3 OMVs, independently of LPS chemotype are able to activate complement *via* all three canonical pathways. Confirming the results of Bjerre et al. (2002), we demonstrated that OMVs possess a higher potential to activate complement than parental bacterial cells or isolated LPS. The essential novelty of our work is the clear demonstration of C’ activation *via* the lectin pathway, suggested by Bjerre et al. (2002). This was induced by a high molecular weight structure resistant to heat, low pH, DNase, and proteinase K. This entity is a target for human and murine MBLs and now is under investigation to establish its structure.

After demonstrating the significant potential of OMVs to activate human complement *in vitro*, we investigated its contribution to the development of the systemic inflammatory response. Previously, a higher capacity of *E. coli* OMVs compared with homologous LPS to induce SIRS symptoms *in vivo* was reported by Park et al. (2010). Similarly, Mirlashari et al. (2001) demonstrated, in whole blood model, higher efficacy of *N* *eisseria meningitidis* vesicles than LPS to stimulate expression of pro‐inflammatory cytokines. However, to our knowledge, the consequences of OMVs‐complement interaction *in vi* *v* *o* haven't been investigated so far. We compared the effect of OMVs in normal and complement‐depleted mice. We found that the intraperitoneal OMVs administration led to altered blood cell counts and up‐regulation of pro‐inflammatory cytokine gene expression. Those differences may be associated with complement activation, since the treatment of mice with CVF resulted in an attenuated (but not totally abolished) response to OMVs. However, it should be taken into account that *Naja naja kaouthia* CVF administration leads not only to C3 depletion but also to formation of C’ activation products (C3a, C5a, sC5b‐9, Bb) which may drive the OMVs‐independent inflammatory response. Additionally, CFV treatment of mice was associated with an increase of hepatic C3 gene expression already at T6, however it was not reflected by native protein C3 detection in serum. It is in agreement with data presented by Younger et al. (2003), who observed serum (and bronchoalveolar lavage fluid, BALF) C3 protein recovery 96 h after induced decomplementation. The involvement of an additional, complement‐independent mechanism(s) in SIRS cannot however be excluded [*e.g*., the lipid A‐associated direct immune cell activation *via* myeloid differentiation protein‐2/Toll‐like receptor 4/CD14 (MD‐2/TLR4/CD14) complex (Lee et al. 2018) as well as *via* intracellular non‐canonical inflammasome, activation (Vanaja et al. 2016)]. The primary descriptive nature of our findings is a limitation to some extent, suggesting that additional research is necessary to investigate their mechanisms.

We reported here for the first time that YeO3 OMVs administration was associated with significantly reduced serum MBL‐C levels in both control and CVF‐treated groups of mice. Similarly, Liu et al. (2001) found a decrease of MBL‐C and increase of MBL‐A after treatment of mice with LPS. MBL‐C is considered as important innate immunity factor (Bidula et al. 2013, Chang et al. 2010). High extrahepatic expression of MBL‐C in the intestine (Wagner et al. 2003) may support its role in the inhibition of the alimentary tract colonization by *Shigella flexneri* 2a (Zuo et al. 2009) and suggests a role in mucosal innate immunity (Uemura et al. 2002). It is possible that the interaction between intestinal MBL‐C (or its human homologue, MBL) with OMVs is involved in *Yersinia*‐induced gastroenteritis.

Our further analysis focused on the biodistribution of *i.p*. administered OMVs, as their contribution to the development of SIRS may depend on their migration in the body, accumulation in organs and clearance rate affected by the length of LPS polysaccharide and deposited products of complement activation. During YeO3 infection, after colonization of intestinal Peyer's patches, bacteria can spread *via* the lymph and/or blood into the mesenteric lymph nodes and can be detected in the liver, spleen, kidneys and lungs. The same could happen to OMVs, which are small enough to move easily and cross body barriers. Our results indicated that YeS‐c_37°C OMVs translocated from the murine peritoneum to the circulation. The partitioning of OMVs between plasma components and blood cells immediately after administration seems to be possible. Like bacterial cells (Minasyan 2016), the recognition and engulfment of OMVs in blood is rather limited due to high velocity of the blood flow and low number of leukocytes. However, Schaack et al. (2024) demonstrated an association of *E. coli* OMVs with blood monocytes after 24 h incubation *in vitro*. Although O'Donoghue et al. (2017) found that OMVs secreted by bacteria with long OPS chains were endocytozed more rapidly and efficiently by epithelial cells than OMVs secreted by bacteria with LPS reduced to the lipid A‐core, we found that OMVs with truncated LPS were quickly cleared from the circulation, compared with those from YeS‐c_37°C. This finding is in agreement with clearance patterns found for LPS: endotoxins of rough chemotypes (Ra and Re) were cleared much faster than those of S type (Hasunuma et al. 2001). The rapid removal of OMVs secreted by rough mutants could be associated with complement: opsonization with C3 activation products and enhanced endocytosis or direct lysis, not hindered by long OPS chains.

We noticed that livers and spleens were the main organs of sequestration of *i.p*. injected OMVs, independently of LPS chemotype. It is in line with the statement that the liver functions as a “firewall” responsible for clearance of bacteria and LPS from the circulation (Balmer et al. 2014). Moreover, several research groups have found the liver to be the main organ responsible for accumulation of OMVs of different origin (Jang et al. 2015; Jones et al. 2020; Park et al. 2010; Seyama et al. 2020). Sinusoidal liver endothelial cells (SLEC), Kupffer's cells and hepatocytes may be involved in the rapid elimination of OMVs from blood (Broadley et al. 2016; Kumar et al. 2024; Smedsrød et al. 2009; Yao et al. 2016). Interestingly, Parent (1990) reported the presence of lectin‐like receptors on rat hepatocytes, able to recognize the IC region in both LPS with long OPS or LPS reduced to the lipid A‐core region. Their murine homologues, with high affinity for heptose residues may be responsible for the especially rapid clearance of YeRd1‐c OMVs from the blood. Interestingly, we were able to detect OMV‐associated fluorescence in gallbladders, suggesting their biliary clearance. Furthermore, we found a significant decrease of concentration of both forms of murine MBL in bile, after OMVs administration. For LPS it is known that chylomicrons and especially high‐density lipoprotein (HDL) are useful in LPS redirection from Kupffer's cells to parenchymal liver cells and enhance its subsequent secretion into the bile (Read et al. 1993). So far, no reports concerning OMVs clearance with bile, OMV‐HDL interactions or OMVs‐induced changes in MBL levels in bile were presented.

OMV sequestration outside the liver and spleen was variable. Accumulation in the lungs was LPS chemotype‐dependent. No YeRe‐c particles were sequestered in the lungs, OMVs with LPS reduced to lipid A‐inner/outer core was detectable already at T3, whereas S‐type OMVs were found in lung tissue at T6–T12 only. Similar findings were presented for *Acinetobacter baumannii* OMVs (Marion et al. 2019). The detection of OMVs in the lung may indicate their role in the induction of lung inflammation and injury. In acute inflammatory states, associated with massive accumulation of inflammatory cells in the lung capillaries, lungs but not livers are the dominant reticuloendothelial system (RES) organs in humans and rodents, responsible for the rapid clearance of foreign particles. As demonstrated by Ryu et al. (2023) and Lee et al. (2018), the presence of OMVs in lung was associated with severe local inflammation.

In the kidney, OMVs (except for Re chemotype) were detected already three hours after administration. Schaack et al. (2024) described the presence *E. coli* OMVs in the kidney even within 1 h after injection. Like Jang et al. (2015), we were unable to detect OMV‐associated fluorescence in the urine of mice treated with YeS‐c_37°C or YeRe‐c_37°C OMV, collected at T6.

OMVs produced by rough type bacteria were detected in inguinal lymph nodes. Interestingly pathogenic *Yersinia* bacteria show a marked tropism to lymphoid tissues during the early stages of infection with the potential to disseminate to deeper tissues of the RES (Young and Miller 1997). Due to that, both lymph nodes and omental milky spots are possibly involved in OMVs uptake from the peritoneum.

The success of OMVs as complex virulence factors and their contribution to SIRS may be affected by their quantity, size, morphology, expression of the surface structures and cargo. These parameters may influence *i.a*. dissemination of the vesicles within the body, consumption of antibodies and innate immunity factors, leading to inflammation and consequently modulating host response to infection. As the expression of numerous virulence factors of YeO3 is temperature‐regulated, we investigated an impact of growth temperature on OMV biogenesis. We found that growth temperature determines the YeO3‐c OMV secretion, with the highest production at 37°C (Table 3). There have been several previous reports concerning the impact of growth temperature on OMV biogenesis, but the results were inconsistent and seem to be species‐dependent. Ma et al. (2022) reported the highest production of OMVs at 15°C and the lowest at 37°C for *Y. enterocolitica* wild type strain Y1083. Other studies reported higher temperature associated with increased (Eddy et al. 2014; McBroom and Kuehn 2007), decreased (Frias et al. 2010; McMahon et al. 2012; Roden et al. 2012) OMV secretion or to have no effect (MacDonald and Kuehn 2013; Taheri et al. 2019). Moreover, it was shown by others that OMV production was enhanced by cold shock in *Y. pestis* (Eddy et al. 2014) but by heat shock in *E. coli* (Katsui et al. 1982).

Analysis of the size distribution of OMVs revealed that the population of OMVs secreted by YeS‐c bacteria grown at 4°C was enriched with larger particles when compared with bacteria cultivated at 37°C. It may suggest that the release of smaller OMVs driven by temperature shift from the environmental to that of host body is a form of OMVs adaptation to play a role of virulence factor. The size of OMVs affects their biological activity, including ability to activate the complement system. Whereas the vesicles sized 40–250 nm were potent activators, those < 30 nm or > 600 nm appeared much less effective (reviewed by La‐Beck et al. 2021). Moreover, Turner et al. (2018) reported that OMVs internalization by host cells depends on their size: OMVs smaller than 100 nm were uptaken *via* caveolin‐mediated endocytosis, whereas larger OMVs were taken up by micropinocytosis.

OMV secretion might be also affected by mutations of the genes associated with synthesis of cell envelope constituents, such as LPS, ECA or PG (Kulp et al. 2015; Liu et al. 2016; McMahon et al. 2012; Pérez‐Ortega et al. 2022; Schwechheimer et al. 2014). Since LPS is the main surface component of the OMVs, affecting the net surface electric charge, OM fluidity and curvature of particles, we investigated also the role of LPS chemotype in OMVs secretion. Our results demonstrating that OMV release was associated with the LPS chemotype supported that conclusion. The highest OMV production was found for YeO3 Rd1‐c variant with LPS truncated to the lipid A‐IC (Table 3). Likewise, Kulp et al. (2015) reported that gene mutations associated with biosynthesis and transfer of heptoses in the IC were associated with increased OMV secretion. However, with *Salmonella enterica* serovar Typhimurium, it was demonstrated that in deep‐rough mutants (LPS of Re or Rd chemotype) the secretion of OMVs was reduced, whereas in mutants with complete (Ra chemotype) or incomplete OC region (Rb2 chemotype) LPS, the vesiculation was enhanced (Liu et al. 2016). It implies that the effect of LPS chemotype on OMVs secretion is species‐dependent, like the impact of growth temperature.

OMVs secretion may be significantly affected by environmental conditions. This issue is well documented for *S. enterica* serovar Typhimurium in the context of PagC, PagL and PagP proteins, activated by the PhoP‐PhoQ system under acidic pH of lysosomes. For example, Dehinwal et al. (2024), demonstrated the increased OMVs secretion in the result of protonation of PagC extracellular loops, modification of its interaction with surrounding LPS (without effect on LPS structure) and altered OM curvature. On the other hand, it can be also associated with a modified lipid A acylation pattern. Elhenawy et al. (2016) found enhanced *Salmonella* OMVs production associated with lipid A deacylation, caused by the overexpression of PagL enzyme and proposed that the accumulation of deacylated lipid A forms with cylindrical/inverted‐cone shape may also modify membrane curvature facilitating the vesiculation process. This phenomenon was clearly visible only for recombinant mutants characterized by overexpression of PagL. Bonnington and Kuehn (2016; 2017) described that at low pH, LPS decorating pathogenic *S. enterica* OMVs is enriched in heptaacylated lipid A and postulated that OMVs are the bacterial way to get rid of disadvantageous LPS glycoforms. Again, the observed phenomenon may be species‐dependent: mutations of genes responsible for acyltransferases involved in lipid A synthesis showed the negative effect on OMVs production in *E. coli* (Kulp et al. 2015) and no differences in amount (or size) between OMVs secreted by *Helicobacter pylori* synthesising LPS with hexa‐ and tetra‐acylated lipid A was observed (Liu et al. 2025).

Our analysis of the effect of growth temperature on OMVs secretion by YeO3 bacteria also indicates the possible association of OMVs release with lipid A acylation pattern: bacteria grown at host's body temperature (37°C) secreted OMVs enriched in tetraacylated lipid A, whereas bacteria cultivated at low environmental temperature at 4°C produced OMVs with hexaacylated lipid A, associated with reduced OM fluidity. The OMVs lipid A of bacteria propagated at 22°C is a mixture of both tetra‐ and hexaacylated lipid A forms. Our results are in agreement with the findings of Fernandez‐Carrasco et al. (2025), who observed that at 37°C, pathogenic but not non‐pathogenic *Yersinia* switch to deacylated lipid A. The lipid A deacylation at 3’‐position resulting in tetraacylated LA I form in Ye is conducted by LpxR lipid A deacylase (Reinés et al. 2012b). Its expression, like that of other virulence factors of Ye, is temperature regulated, with highest synthesis at 37°C and therefore may affect the acylation pattern of OMVs at host body temperature. Our chemical analysis of cell‐ and OMV‐associated lipid A confirmed structures of YeO3 cell‐derived lipid A published by others (Fernandez‐Carrasco et al. 2025; Pérez‐Gutiérrez et al. 2010; Rebeil et al. 2004; Reinés et al. 2012a; Reinés et al. 2012b). Contrary to Elhenawy et al. (2016) and Haurat et al. (2011), we did not observe differences in the acylation pattern of lipid A between cell‐ and OMVs‐associated LPS: no increased accumulation of deacylated lipid A form exclusively in OMVs was observed comparing ions intensity.

The acylation pattern of OMVs‐associated lipid A may affect its biological activity. In agreement with data reported for isolated LPS (Rietschel et al. 1994), our findings of temperature shift‐induced removal of acyl groups may suggest lower potential of Ye OMVs of bacteria propagated at 37°C to host's cell stimulation. In contrast, Wiese et al. (2001) found that the higher fluidity of model endotoxin/phospholipid bilayers containing *E. coli* pentaacylated LPS (when compared with hexaacylated LPS containing bilayers) was correlated with higher level of complement activation, whereas Liu et al. (2025) observed higher antibody production in response to OMVs secreted by bacteria with hexa‐ than tetraacylated lipid A. However, it is not the structure of lipid A that contributes to the highest number of secreted crude OMVs in the case of the Rd1 mutant, since negligible differences were observed in lipid A structure for Ra, Rd1, and Re strains (Table 4, Figure S6).

Our findings draw attention to the OMVs as potent activators of the complement system, which may lead to life‐threatening systemic host response both in acute or persistent infections and perhaps also in patients with increased permeability of intestinal barrier not associated with infection. They provide a strong rationale for introducing complement inhibition‐based therapies to reduce consequences of OMVs‐related toxicity. Although yersiniosis is not the priority disease for vaccine development, our findings highlight the necessity to consider complement activation in the design of OMVs‐based vaccines. On the one hand, deposition of C’ activation products may significantly increase their immunogenicity due to the strong adjuvant potential of complement C3d (Dempsey et al. 1996). But on the other hand—complement activation may lead to the development of post‐vaccination complications after administration of preparations based on extracellular vesicles. These complications may be additionally related to the toxicity of OMVs‐associated lipid A. It seems that a solution of the latter problem is development of low‐toxic vaccines, with modified lipid A acylation (Liu et al. 2025; Majumder et al. 2024). Moreover, our findings concerning the association of LPS chemotype with OMVs secretion may be essential for OMVs‐based vaccine production. In addition, the biodistribution data demonstrated possible spreading and clearance routes of OMVs, which may be important in vaccine development.

Our results add considerably to the knowledge concerning the impact of growth conditions (temperature) and LPS structure on production of OMVs. As carriers of a variety of virulence factors, including endotoxin, they are able to modulate host immune responses as demonstrated by their influence on serum bactericidal activity, ability to activate complement *via* all canonical pathways, to induce symptoms of systemic inflammation and influence migration in the body. Furthermore, we detected high‐molecular weight glycoconjugate, distinct from LPS (and present in YeO3 OMVs independently of LPS chemotype), being recognized by human and murine MBLs resulting in C’ activation *via* the lectin pathway. The mechanism of its incorporation into OMVs and its role is undoubtedly interesting and warrants further investigation. It may be supposed that it contributes to the evasion of the immune response by pathogen through consumption of the MBL‐MASP complex, and consequently C4, C3, C5 factors, thus preventing formation of complement convertases and TCC on bacterial cell envelope.

## Author Contributions

**Cédric Battaglino**: investigation, methodology, writing – review and editing, formal analysis, visualization. **Iryna Bodnaruk**: investigation, methodology, writing – review and editing, formal analysis, visualization. **Paula Czyszczoń**: investigation, writing – review and editing, visualization, methodology, formal analysis. **Mikael Skurnik**: writing – review and editing, methodology. **Beata Filip‐Psurska**: investigation, validation, methodology, writing – review and editing, formal analysis. **Dariusz Jarych**: investigation, methodology, writing – review and editing, formal analysis. **Anna Maciejewska**: investigation, writing – review and editing, validation, visualization, formal analysis. **Mariusz Gadzinowski**: investigation, methodology, writing – review and editing. **Kamil Malik**: investigation, visualization, writing – review and editing. **Izabela Potocka**: investigation, visualization, formal analysis, writing – review and editing. **Paweł Migdał**: investigation, visualization, writing – review and editing, formal analysis. **Roksana Kruszakin**: investigation, validation, visualization, formal analysis, writing – review and editing. **Maciej Cedzyński**: conceptualization, writing – original draft, validation, visualization, writing – review and editing, formal analysis, supervision, data curation, funding acquisition. **Katarzyna Kasperkiewicz**: conceptualization, investigation, funding acquisition, writing – review and editing, writing – original draft, methodology, visualization, formal analysis, project administration, supervision, data curation, validation. **Jolanta Lukasiewicz**: conceptualization, investigation, funding acquisition, writing – original draft, methodology, validation, visualization, writing – review and editing, formal analysis, project administration, data curation, supervision. **Anna S. Świerzko**: conceptualization, investigation, funding acquisition, writing – original draft, methodology, validation, visualization, writing – review and editing, formal analysis, project administration, supervision, data curation.

## Funding

This work was supported by National Science Centre, Poland, grant 2018/31/B/NZ6/03514.

## Ethical Approval Statement

Approvals of the local ethical committee were obtained.

## Conflicts of Interest

The authors report no conflicts of interest.

## Supporting information




**Supplementary Figure 1**. Electron microscopy‐based detection of OMVs secreted by *pYV*‐cured *Yersinia enterocolitica* O:3 bacteria. **Supplementary Figure 2**. Enterobacterial common antigen (ECA) detection on OMVs and bacterial cells. **Supplementary Figure 3**. Inhibition of lytic activity of *Yersinia enterocolitica* O:3 OPS‐specific bacteriophage. **Supplementary Table 1**. Analysis of *Y. enterocolitica* O:3 OMVs size distribution. **Supplementary Figure 4**. Chromatograms of soluble supernatants obtained by mild acid hydrolysis (1.5% CH_3_COOH) of LPSs (100 µg, **A‐C**) and OMVs (23‐71 µg, **D‐F**) of *Y. enterocolitica* O:3 (S chemotype) cultivated at 37°C, 22°C, and 4°C. **Supplementary Figure 5**. Comparative ^1^H NMR analysis of the OPSs fractions in cell‐derived LPS and OMV‐derived LPS isolated from *Y. enterocolitica* O:3 S cultured at 37°C. **Supplementary Figure 6**. Comparison of MALDI‐TOF mass spectra obtained in negative ion mode for cell‐derived LPS and OMV‐derived LPS isolated from *Y. enterocolitica* O:3 S, Ra, Rd1, and Re cultivated at 37°C. **Supplementary Table 2**. Major forms of lipids A identified by MALDI‐TOF mass spectrometry in *Y. enterocolitica* O:3 cell‐derived LPS and OMV‐derived LPS. **Supplementary Figure 7**. Interaction of human serum MBL, ficolin‐1, ficolin‐2 and ficolin‐3 with crude YeS‐c_37°C (1), YeRa‐c_37°C (2), YeRd1‐c_37°C (3) and YeRe‐c_37°C (4). 10 µl of OMVs suspensions was spotted on nitrocellulose membrane. **Supplementary Figure 8**. Recognition of *Yersinia enterocolitica* O:3 OMVs by murine MBL‐A and MBL‐C. **Supplementary Figure 9**. Western blot analysis of mannose‐binding lectin (MBL) interaction with *Yersinia* OMVs. OMVs (20 µl) were separated in SDS‐PAGE and transferred to nitrocellulose membrane. **Supplementary Figure 10**. Detection of C3 α chain in sera of native (0.9% NaCl‐treated) mice or mice with induced decomplementation (CVF‐treated). **Supplementary Figure 11**. The comparison of fluorescence in organs of mice treated with 0.9% NaCl and DiD‐NaCl in analyzed groups. **Supplementary Figure 12**. Detection of *Yersinia enterocolitica* O:3‐reactive antibodies in normal human serum. **Supplementary Figure 13**. The effect of *E. coli* O:16 and *K. pneumoniae* OMVs on bactericidal activity toward YeS‐c bacteria.

## Data Availability

All data related to the study are available from the corresponding author upon reasonable request.
