# Supplementary material for: Yersinia enterocolitica O:3 Outer Membrane Vesicles as a Platform for Complement Activation
Source: J Extracell Vesicles. 2026 Apr 17;15(4):e70270. doi: 10.1002/jev2.70270 (PMC13088890; doi:10.1002/jev2.70270)
Supplement: Supplementary file 1 — Supplementary Figure 1. Electron microscopy‐based detection of OMVs secreted by pYV‐cured Yersinia enterocolitica O:3 bacteria. Supplementary Figure 2. Enterobacterial common antigen (ECA) detection on OMVs and bacterial cells. Supplementary Figure 3. Inhibition of lytic activity of Yersinia enterocolitica O:3 OPS‐specific bacteriophage. Supplementary Table 1. Analysis of Y. enterocolitica O:3 OMVs size distribution. Supplementary Figure 4. Chromatograms of soluble supernatants obtained by mild acid hydrolysis (1.5% CH3COOH) of LPSs (100 µg, A‐C) and OMVs (23‐71 µg, D‐F) of Y. enterocolitica O:3 (S chemotype) cultivated at 37°C, 22°C, and 4°C. Supplementary Figure 5. Comparative 1H NMR analysis of the OPSs fractions in cell‐derived LPS and OMV‐derived LPS isolated from Y. enterocolitica O:3 S cultured at 37°C. Supplementary Figure 6. Comparison of MALDI‐TOF mass spectra obtained in negative ion mode for cell‐derived LPS and OMV‐derived LPS isolated from Y. enterocolitica O:3 S, Ra, Rd1, and Re cultivated at 37°C. Supplementary Table 2. Major forms of lipids A identified by MALDI‐TOF mass spectrometry in Y. enterocolitica O:3 cell‐derived LPS and OMV‐derived LPS. Supplementary Figure 7. Interaction of human serum MBL, ficolin‐1, ficolin‐2 and ficolin‐3 with crude YeS‐c_37°C (1), YeRa‐c_37°C (2), YeRd1‐c_37°C (3) and YeRe‐c_37°C (4). 10 µl of OMVs suspensions was spotted on nitrocellulose membrane. Supplementary Figure 8. Recognition of Yersinia enterocolitica O:3 OMVs by murine MBL‐A and MBL‐C. Supplementary Figure 9. Western blot analysis of mannose‐binding lectin (MBL) interaction with Yersinia OMVs. OMVs (20 µl) were separated in SDS‐PAGE and transferred to nitrocellulose membrane. Supplementary Figure 10. Detection of C3 α chain in sera of native (0.9% NaCl‐treated) mice or mice with induced decomplementation (CVF‐treated). Supplementary Figure 11. The comparison of fluorescence in organs of mice treated with 0.9% NaCl and DiD‐NaCl in analyzed groups. Supplementa [file JEV2-15-e70270-s001.docx]

**Supplementary materials**

***Yersinia enterocolitica* O:3 outer membrane vesicles as a platform for complement activation**

Cédric Battaglino, Iryna Bodnaruk, Paula Czyszczoń, Mikael Skurnik, Beata Filip-Psurska, Dariusz Jarych, Anna Maciejewska, Mariusz Gadzinowski, Kamil Malik, Izabela Potocka, Paweł Migdał, Roksana Kruszakin, Maciej Cedzyński, Katarzyna Kasperkiewicz, Jolanta Lukasiewicz, Anna S. Świerzko


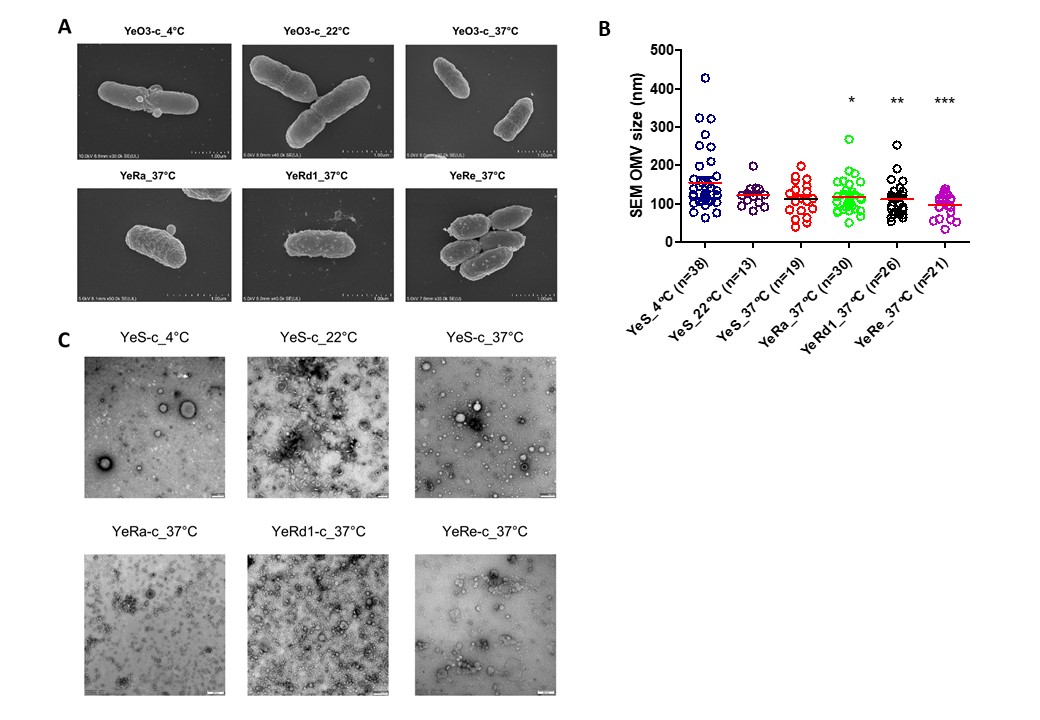


**Supplementary Figure 1.** Electron microscopy-based detection of OMVs secreted by *pYV*-cured *Yersinia enterocolitica* O:3 bacteria. **A:**  SEM visualization of OMVs in culture supernatants at 2 µm scale; **B:** analysis of OMVs dimension. Mann-Whitney test was used for statistical analysis. * *p*<0.05; ** *p*<0.001; *p*<0.0001; **C:** TEM analysis of density gradient-purified OMVs at 200 nm scale.


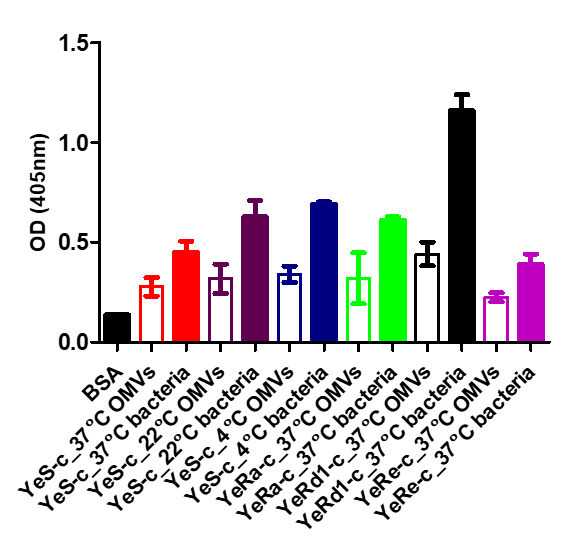


**Supplementary Figure 2**. Enterobacterial common antigen (ECA) detection on OMVs and bacterial cells. Wells of microtiter plates were coated with 50 ng of OMVs or bacterial cells. After blocking, ECA was detected with specific monoclonal and corresponding secondary antibodies, as described in Material and Methods section.


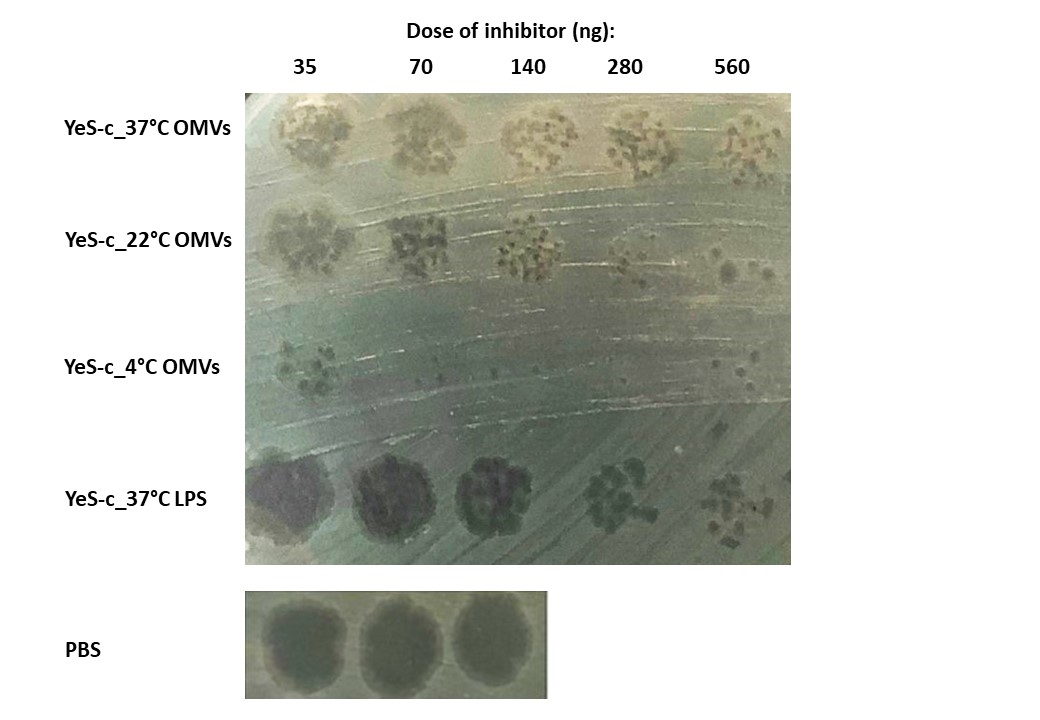


**Supplementary Figure 3.** Inhibition of lytic activity of *Yersinia enterocolitica* O:3 OPS-specific bacteriophage. Phage ɸYeO3-12 was preincubated with YeO3-c_37°C LPS or YeO3 OMVs secreted by bacteria grown at 4°C, 22°C or 37°C (as inhibitors) and serially diluted before being spotted on YeO3 bacterial lawn.

**Supplementary Table 1.** Analysis of *Y. enterocolitica* O:3 OMVs size distribution.^a^

| Strain/growth temperature | OMVs concentration (**×**10^10^) depending on size range | | | | | | | | |
| --- | --- | --- | --- | --- | --- | --- | --- | --- | --- |
|  | 50-199 nm | | | 100-199 nm | | | 100-299 nm | | |
|  | <100 | ≥100 | p | <150 | ≥150 | p | <150 | ≥150 | p |
| YeS-c_4°C | 0.22 | 3.2 | <0.0001 | 1.4 | 1.8 | 0.04 | 1.4 | 3.4 | 0.03 |
| YeS-c_22°C | 0.14 | 3.4 | <0.0001 | 1.7 | 1.7 | 0.129 | 1.7 | 2.8 | <0.0001 |
| YeS-c_37°C | 1.8 | 14.1 | <0.0001 | 9.2 | 4.7 | <0.0001 | 9.2 | 8.3 | <0.0001 |
| YeRa-c_37°C | 0.36 | 4.1 | <0.0001 | 2.7 | 1.4 | <0.0001 | 2.7 | 2.3 | <0.0001 |
| YeRd1-c_37°C | 3.1 | 12.1 | <0.0001 | 7.1 | 5.3 | <0.0001 | 7.1 | 9.0 | <0.0001 |
| YeRe-c_37°C | 0.29 | 3.8 | <0.0001 | 2.8 | 1.0 | <0.0001 | 2.8 | 1.7 | <0.0001 |

^a^ non-parametric Mann-Whitney *U*  test was used.


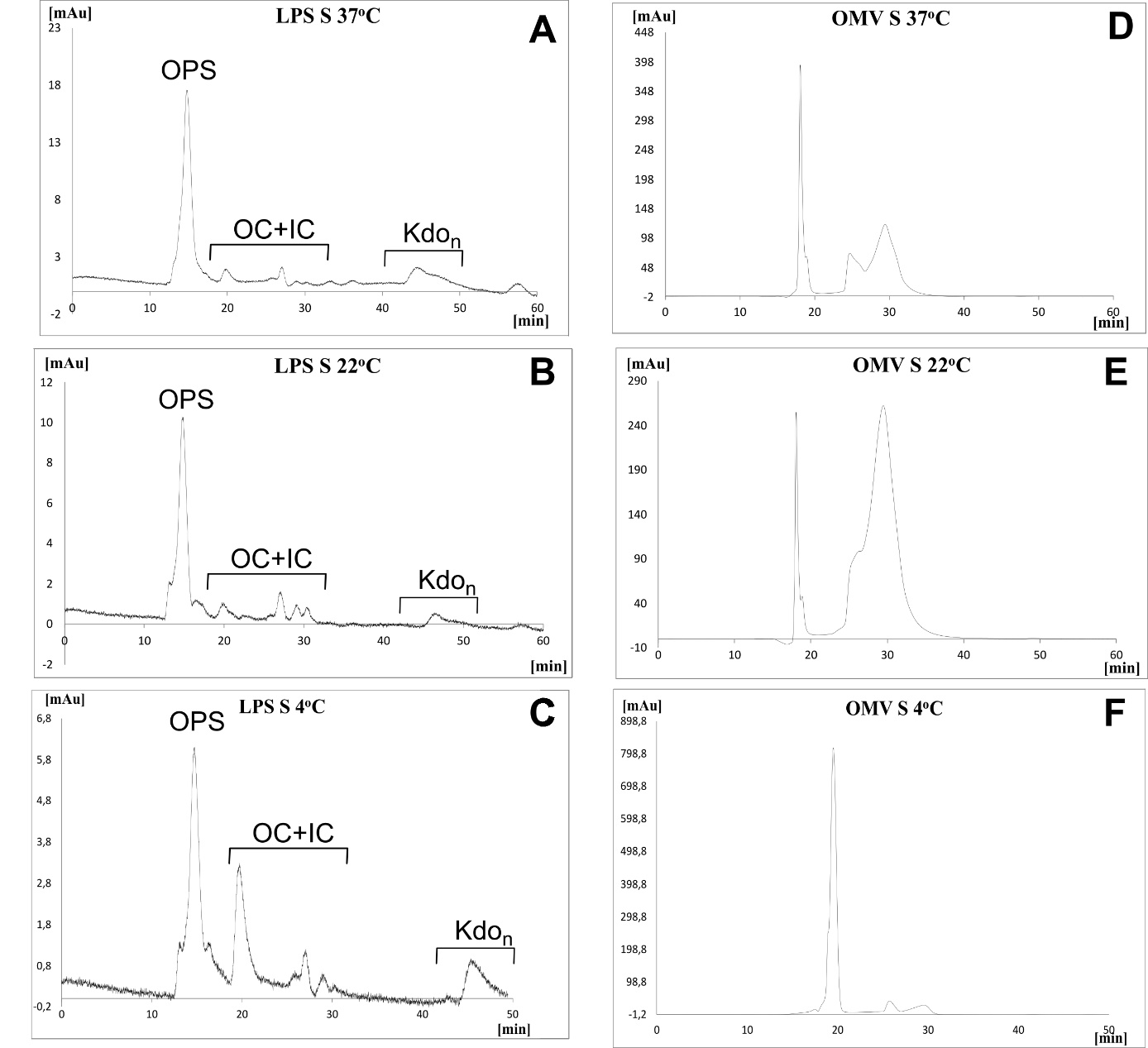


**Supplementary Figure 4.** Chromatograms of soluble supernatants obtained by mild acid hydrolysis (1.5% CH_3_COOH) of LPSs (100 µg, **A-C**) and OMVs (23-71 µg, **D-F**) of *Y. enterocolitica* O:3 (S chemotype) cultivated at 37°C, 22°C, and 4°C. Supernatants were fractionated on TSKgel®G2000PW column (flow: 1 ml/min). Chromatograms were monitored by UV absorption at 190 nm. OPS – O-specific polysaccharide fraction; OC – outer core; IC – inner core; Kdo_n_ – Kdo mono- and oligosaccharides released after mild acid hydrolysis.


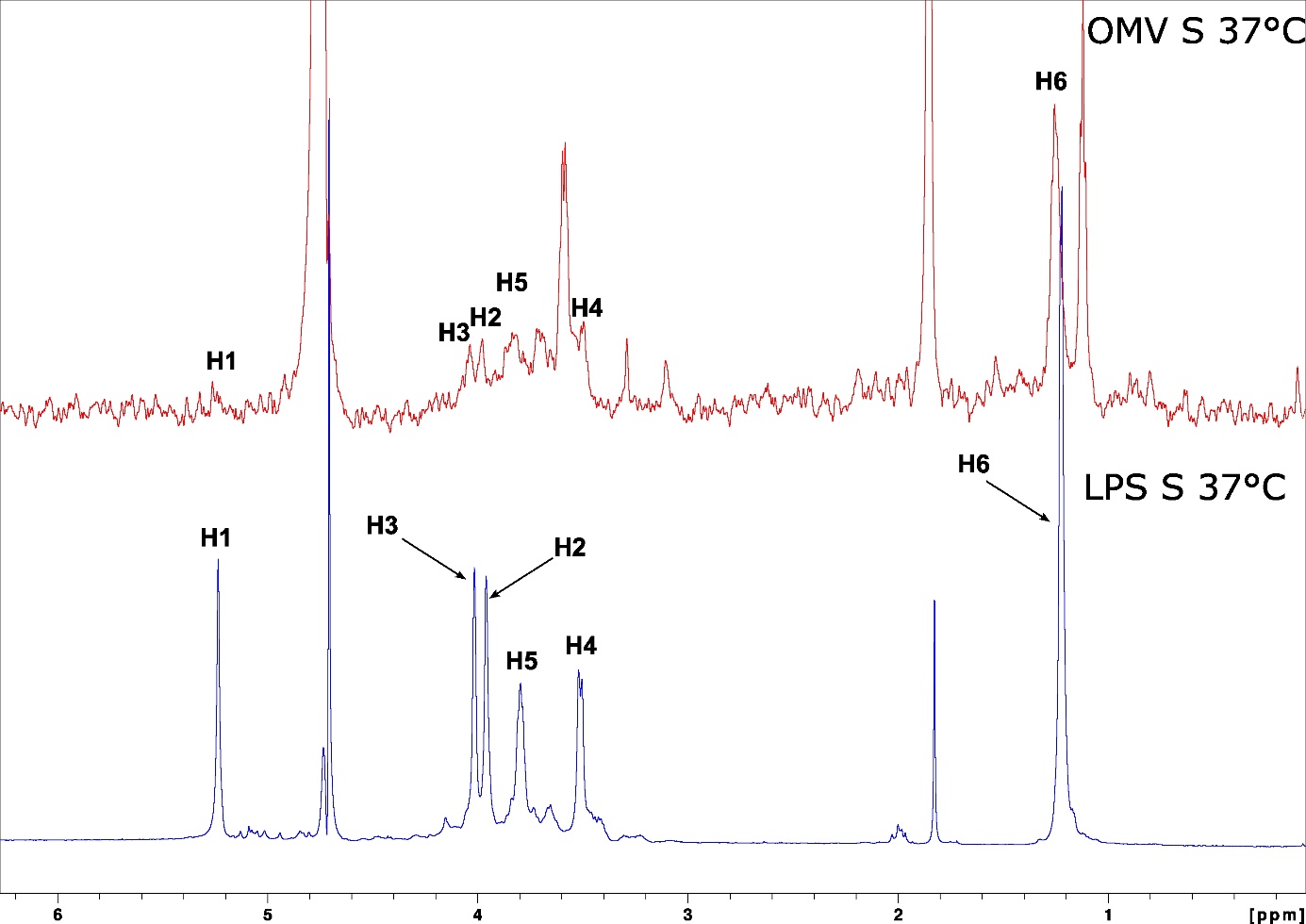
**Supplementary Figure 5.** Comparative ^1^H NMR analysis of the OPSs fractions in cell-derived LPS and OMV-derived LPS isolated from *Y. enterocolitica* O:3 S cultured at 37°C. OPS fractions were isolated as was shown in supplementary Figure 4A, D. Capital letters refer to protons of polysaccharide built of →2)-β-6-deoxy-L-altrose as was shown in Figure 1 and previously published (Muszynski et al., 2013).


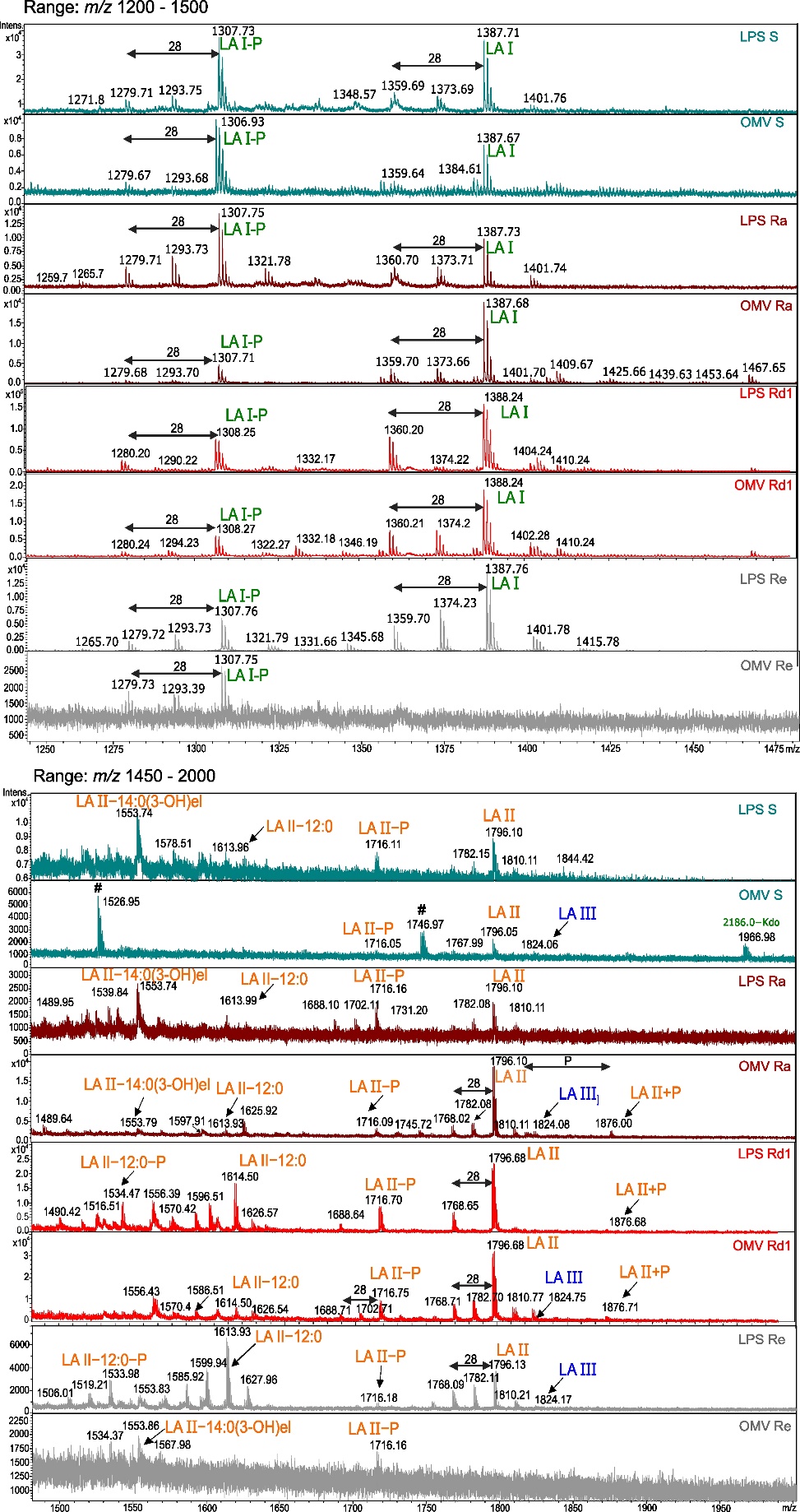


**Supplementary Figure 6.** Comparison of MALDI-TOF mass spectra obtained in negative ion mode for cell-derived LPS and OMV-derived LPS isolated from *Y. enterocolitica* O:3 S, Ra, Rd1, and Re cultivated at 37°C. Upper and lower panels show 1200-1500 and 1450-2000 *m/z* ranges, respectively. LA – lipid A; LA I, II, III – forms of YeO3 lipids A as shown in Figure 3 and explained in supplementary Table 2. 14:0(3-OH) - 3-hydroxytetradecanoic acid; 12:0 – dodecanoic acid, P – phosphate group, el – acyl residue elimination, 28 – mass difference attributed to -CH_2_CH_2_-.

**Supplementary Table 2**. Major forms of lipids A identified by MALDI-TOF mass spectrometry in *Y. enterocolitica* O:3 cell-derived LPS and OMV-derived LPS.^a^

| Ion  [*m/z*] | Lipid A form | Composition | | | | | |
| --- | --- | --- | --- | --- | --- | --- | --- |
|  |  | Lipid A backbone | Acylation pattern and additional substituents | | | | |
|  |  |  | position 2 | position 3 | position 2’ | position 3’ | position 4’ |
| 1307.73 | LA I−P | β-D-Glc*p*N**4P**-(1→6)-β-D-Glc*p*N | 14:0(3-OH) | 14:0(3-OH) | 14:0(3-O14:0) |  |  |
| 1334.56 | LA III−14:0(3-O-12:0)−P | β-D-Glc*p*N**4P**-(1→6)-β-D-Glc*p*N | 14:0(3-OH) | 14:0(3-OH) | 14:0(3-O-**16:1**) |  |  |
| 1387.71 | LA I | β-D-Glc*p*N**4P**-(1→6)-β-D-Glc*p*N**1P** | 14:0(3-OH) | 14:0(3-OH) | 14:0(3-O14:0) |  |  |
| 1516.82 | LA III−14:0(3-OH)−P | β-D-Glc*p*N**4P**-(1→6)-β-D-Glc*p*N | 14:0(3-OH) |  | 14:0(3-O-**16:1**) | 14:0(3-O-12:0) |  |
| 1533.95 | LA II−12:0−P | β-D-Glc*p*N**4P**-(1→6)-β-D-Glc*p*N | 14:0(3-OH) | 14:0(3-OH) | 14:0(3-O-14:0) | 14:0(3-OH) |  |
| 1560.88 | LA III−12:0−P | β-D-Glc*p*N**4P**-(1→6)-β-D-Glc*p*N | 14:0(3-OH) | 14:0(3-OH) | 14:0(3-O-**16:1**) | 14:0(3-OH) |  |
| 1613.96 | LA II−12:0 | β-D-Glc*p*N**4P**-(1→6)-β-D-Glc*p*N**1P** | 14:0(3-OH) | 14:0(3-OH) | 14:0(3-O-14:0) | 14:0(3-OH) |  |
| 1640.88 | LA III−12:0 | β-D-Glc*p*N**4P**-(1→6)-β-D-Glc*p*N**1P** | 14:0(3-OH) | 14:0(3-OH) | 14:0(3-O-**16:1**) | 14:0(3-OH) |  |
| 1716.11 | LA II−P | β-D-Glc*p*N**4P**-(1→6)-β-D-Glc*p*N | 14:0(3-OH) | 14:0(3-OH) | 14:0(3-O-14:0) | 14:0(3-O-12:0) |  |
| 1742.32 | LA III−P | β-D-Glc*p*N**4P**-(1→6)-β-D-Glc*p*N | 14:0(3-OH) | 14:0(3-OH) | 14:0(3-O-**16:1**) | 14:0(3-O-12:0) |  |
| 1796.10 | LA II | β-D-Glc*p*N**4P**-(1→6)-β-D-Glc*p*N**1P** | 14:0(3-OH) | 14:0(3-OH) | 14:0(3-O-14:0) | 14:0(3-O-12:0) |  |
| 1822.26 | LA III | β-D-Glc*p*N**4P**-(1→6)-β-D-Glc*p*N**1P** | 14:0(3-OH) | 14:0(3-OH) | 14:0(3-O-**16:1**) | 14:0(3-O-12:0) |  |
| 1874.24 | LA III+Ara4N−P | β-D-Glc*p*N**4P**-(1→6)-β-D-Glc*p*N | 14:0(3-OH) | 14:0(3-OH) | 14:0(3-O-**16:1**) | 14:0(3-O-12:0) | α-L-Ara4N |
| 1953.49 | LA III+Ara4N | β-D-Glc*p*N**4P**-(1→6)-β-D-Glc*p*N**1P** | 14:0(3-OH) | 14:0(3-OH) | 14:0(3-O-**16:1**) | 14:0(3-O-12:0) | α-L-Ara4N |
| 2034.60 | LAII+16:0 | β-D-Glc*p*N**4P**-(1→6)-β-D-Glc*p*N**1P** | 14:0(3-O-**16:0**) | 14:0(3-OH) | 14:0(3-O-14:0) | 14:0(3-O-12:0) |  |
| 2060.42 | LA III+16:0 | β-D-Glc*p*N**4P**-(1→6)-β-D-Glc*p*N**1P** | 14:0(3-O-**16:0**) | 14:0(3-OH) | 14:0(3-O-**16:1**) | 14:0(3-O-12:0) |  |

^a^ Interpretation based on published structures of *Y. enterocolitica* lipids A. LA – lipid A; LA I, II, III, – forms of YeO3 lipids A; data characteristic for LA I, II, III are coloured in green, orange, and blue. GlcN – D-glucosamine; Ara4N - α-L-aminoarabinose, 16:0 – hexadecanoic acid (palmitic acid), 16:1 - 16:1Δ^9^ – *cis*-hexadec-9-enoic acid; 14:0 – tetradecanoic acid, 14:0(3-OH) - 3-hydroxytetradecanoic acid; 12:0 – dodecanoic acid, P – phosphate group, 28 indicates mass difference of -CH_2_-CH_2_- group. Characteristic substituents and acyl residues are marked in bold font.


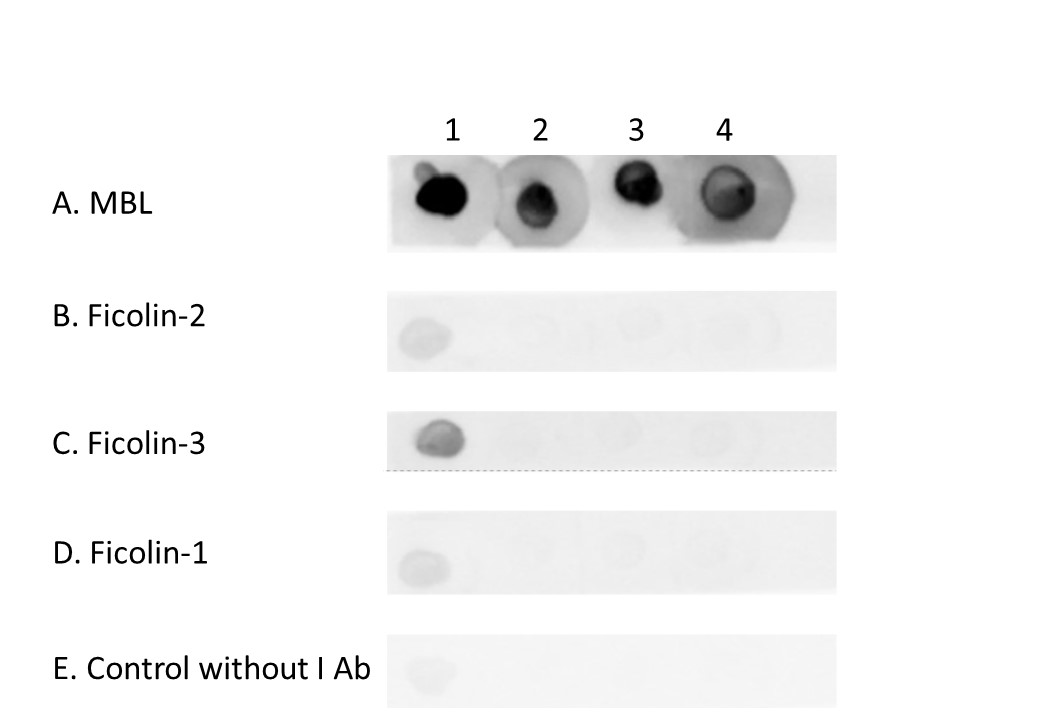


**Supplementary Figure 7**. Interaction of human serum MBL, ficolin-1, ficolin-2 and ficolin-3 with crude YeS-c_37°C (1), YeRa-c_37°C (2), YeRd1-c_37°C (3) and YeRe-c_37°C (4). 10µl of OMVs suspensions was spotted on nitrocellulose membrane. After blocking, membrane A was incubated with high MBL human serum and next - with mouse anti-human MBL (HYB 131-01) mAb, whereas membranes B, C and D were incubated with NHS and anti-ficolin 2 (ABS 05-16, AntibodyShop, anti, ficolin-3 (RIG 334, Invitrogen) or anti- ficolin-1 (ABS 036-1, AntibodyShop) mAbs, respectively. The membrane E was incubated with the mixture of both sera. After incubation with secondary antibodies, the reaction was visualised with ECL method.


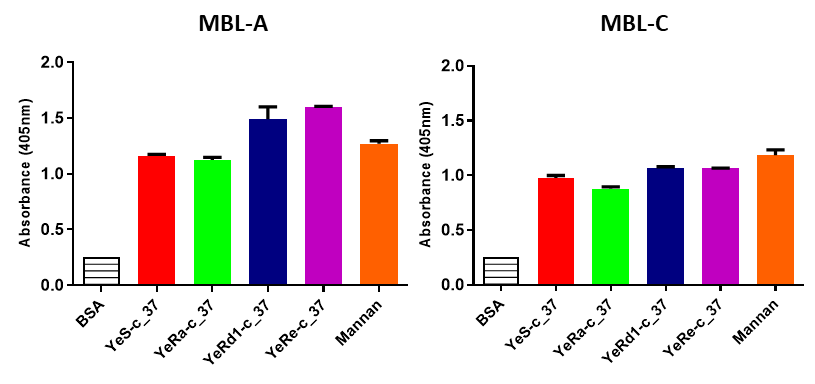


**Supplementary Figure 8**. Recognition of *Yersinia enterocolitica* O:3 OMVs by murine MBL-A and MBL-C. Plates coated with YeS-c_37°C, YeRa-c_37°C, YeRd1-c_37°C, YeRe-c_37°C OMVs (50 ng/well) were incubated with C57BL/6 murine serum. For MBL-A and MBL-C analysis, sera were diluted 500x and 2000x, respectively. Bound MBL-A and MBL-C were detected with specific monoclonal antibodies and corresponding secondary Abs.

**
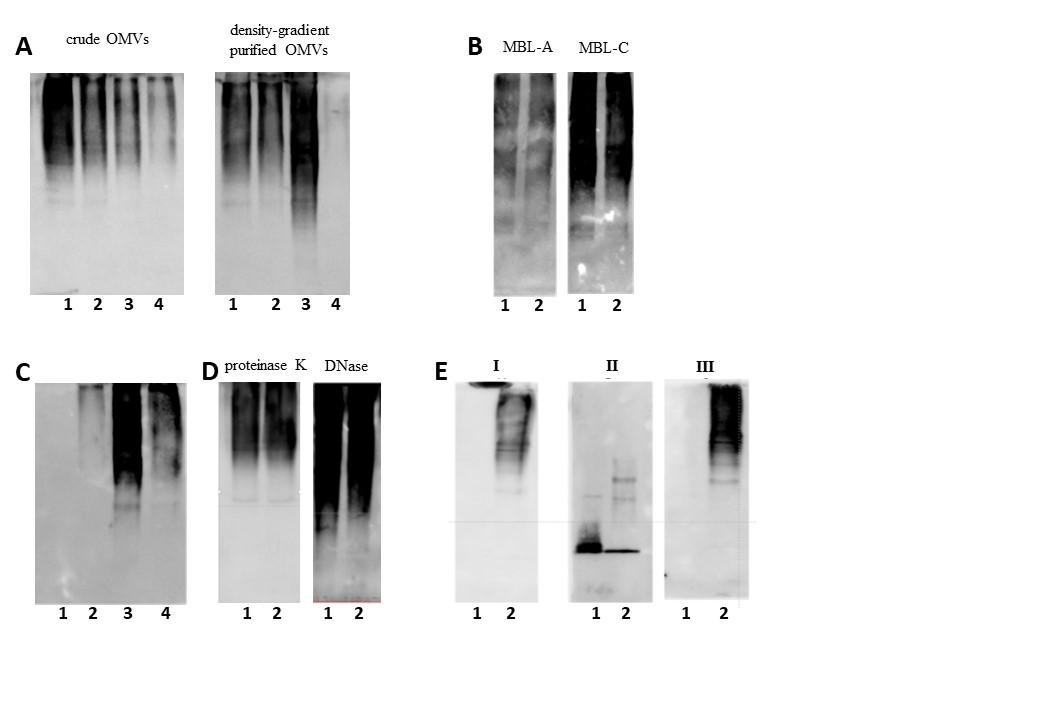
**

**Supplementary Figure 9.** Western blot analysis of mannose-binding lectin (MBL) interaction with *Yersinia* OMVs. OMVs (20 µl) were separated in SDS-PAGE and transferred to nitrocellulose membrane. After incubation with MBL-reach human (A, C, D and E) or murine C57BL serum (B), bound human MBL (A, C, D and E) or murine MBL-A and MBL-C (B) were detected with specific mAbs, respectively.

**A.** Human serum MBL (hMBL) reaction with crude or density gradient purified YeS-c_37°C (1), YeRa-c_37° (2), YeRd1-c_37°C (3), and YeRe-c_37°C (4) OMVs.

**B.** Serum mouse MBL-A and MBL-C reaction with crude or density gradient purified YeS-c_37°C.

**C.** hMBL reaction with vesicles isolated from filtered 500 ml of sterile LB medium (1 and 2, two separate isolations), 500 ml of YeRa-c_37°C sterile spent medium (3) or density gradient purified YeRa-c_37°C (4) OMVs.

**D.** Interaction of hMBL with native (1) and DNase or Proteinase K-treated crude (2) YeS-c_37°C OMVs.

**E.** Human MBL binding to LPS (1) and OMVs (2) expressed by YeS-c_37°C bacteria before (I) and after hydrolysis with 1% CH_3_COOH for 4 min at 100°C (III). The OPS hydrolysis and unmasking of LPS lipid A after treatment with 1%CH_3_COOH was demonstrated with lipid A A6 specific mAb (II).


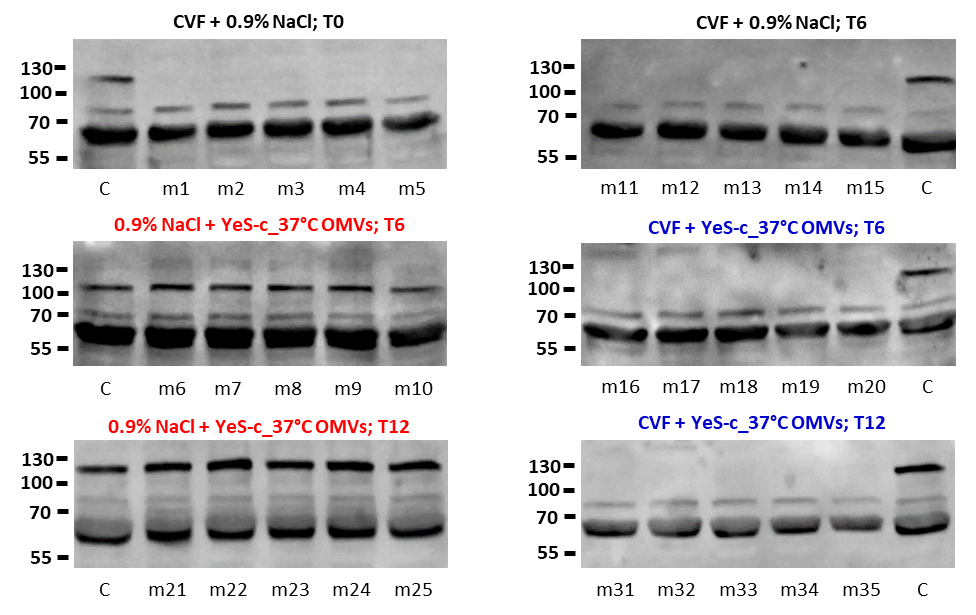


**Supplementary Figure 10**. Detection of C3 α chain in sera of native (0.9% NaCl-treated) mice or mice with induced decomplementation (CVF-treated). C - control serum (taken 28 h before starting treatment with CVF); M1-35-results for individual mice. Mice with induced decomplementation: black description; mice treated with OMVs: red description; mice with induced decomplementation and treated with OMVs: blue description


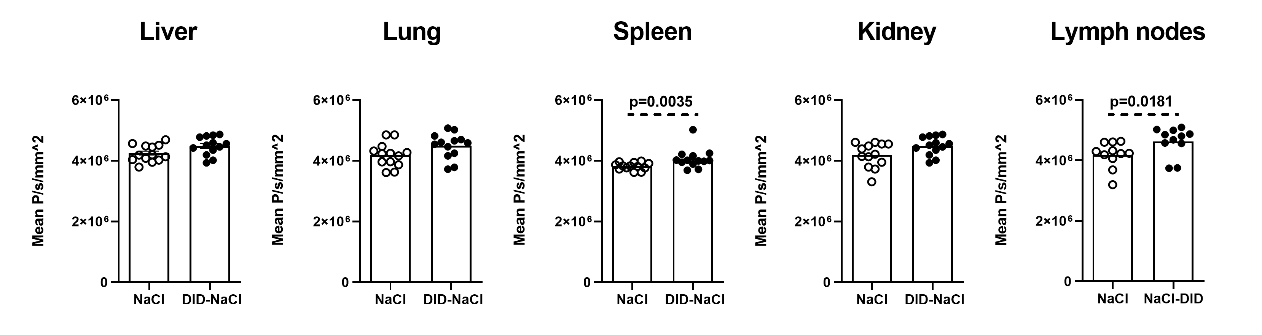


**Supplementary Figure 11**. The comparison of fluorescence in organs of mice treated with 0.9% NaCl and DiD-NaCl in analysed groups.


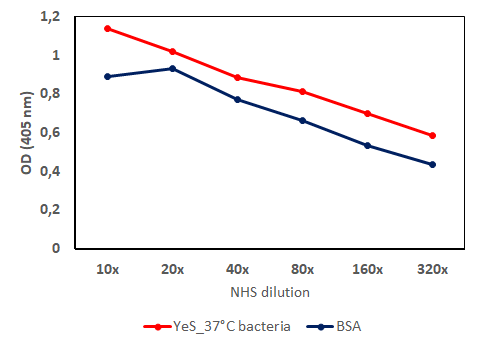


**Supplementary Figure 12**. Detection of *Yersinia entercolitica* O:3-reactive antibodies in normal human serum. Plates were coated with PFA-inactivated YeS-c_37°C bacterial cells (5 µg/well). As a negative control, 1% BSA (blocking agent) was used. After incubation at 37°C, human antibodies bound to OMVs or BSA were detected with HRP-labelled goat anti-human Ig (Dsko).


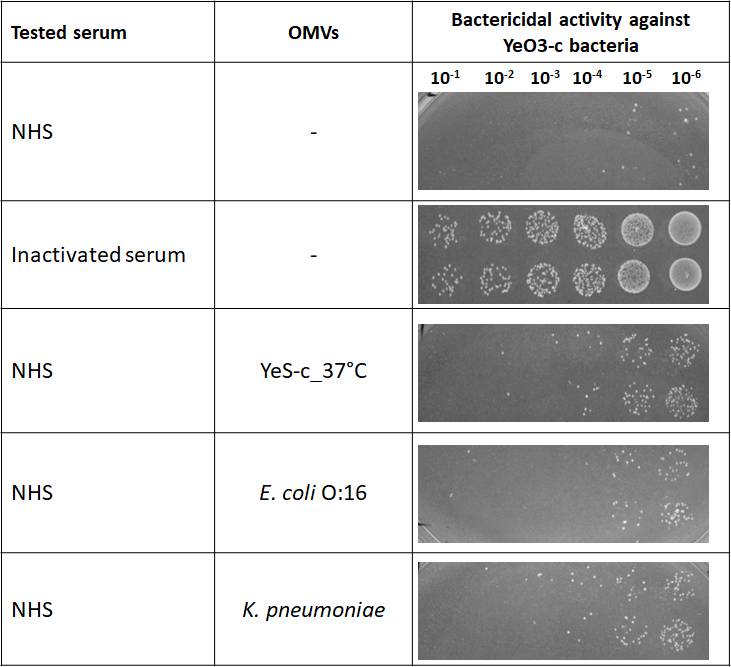


**Supplementary Figure 13.** The effect of *E. coli* O:16 and *K. pneumoniae* OMVs on bactericidal activity towards YeS-c bacteria.
